# Exploring the role of ubiquitin regulatory X domain family proteins in cancers: bioinformatics insights, mechanisms, and implications for therapy

**DOI:** 10.1186/s12967-024-04890-9

**Published:** 2024-02-15

**Authors:** Enyu Yang, Xiaowei Fan, Haihan Ye, Xiaoyang Sun, Qing Ji, Qianyun Ding, Shulian Zhong, Shuo Zhao, Cheng Xuan, Meiyu Fang, Xianfeng Ding, Jun Cao

**Affiliations:** 1 School of Life Sciences and Medicine, Zhejiang Sci-Tech University, Hangzhou, 310018 China; 2 Key Laboratory of Head & Neck Cancer Translational Research of Zhejiang Province, Department of Head and Neck and Rare Oncology, Zhejiang Cancer Hospital, Hangzhou Institute of Medicine (HIM), Chinese Academy of Sciences, Hangzhou, 310022 China; 3 School of Biological Sciences, The University of Hong Kong, Hong Kong , 999077 Special Administrative Region China; 4 Department of ‘A’, The Children’s Hospital, National Clinical Research Center for Child Health, Zhejiang University School of Medicine, Hangzhou, 310003 China; 5 Zhejiang Sci-Tech University Hospital, Zhejiang Sci-Tech University, Hangzhou, 310018 China

**Keywords:** UBXD, Bioinformatic, Cancers, Mechanism, Drugtherapy

## Abstract

UBXD family (UBXDF), a group of proteins containing ubiquitin regulatory X (UBX) domains, play a crucial role in the imbalance of proliferation and apoptotic in cancer. In this study, we summarised bioinformatics proof on multi-omics databases and literature on UBXDF’s effects on cancer. Bioinformatics analysis revealed that Fas-associated factor 1 (FAF1) has the largest number of gene alterations in the UBXD family and has been linked to survival and cancer progression in many cancers. UBXDF may affect tumour microenvironment (TME) and drugtherapy and should be investigated in the future. We also summarised the experimental evidence of the mechanism of UBXDF in cancer, both in vitro and in vivo, as well as its application in clinical and targeted drugs. We compared bioinformatics and literature to provide a multi-omics insight into UBXDF in cancers, review proof and mechanism of UBXDF effects on cancers, and prospect future research directions in-depth. We hope that this paper will be helpful for direct cancer-related UBXDF studies.

**Supplementary Information:**

The online version contains supplementary material available at 10.1186/s12967-024-04890-9.

## Introduction

Ubiquitin is a small protein found in all eukaryotic organisms (most eukaryotic cells). It regulates the function of proteins in a significant way. Ubiquitin disorder can lead to a variety of human diseases [1]. Irwin Allan Rose, Aaron Ciechanover, and Avram Hershko were awarded the 2004 Nobel Prize in Chemistry for their discovery of ubiquitin-regulated protein degradation [2]. UBXD family (UBXDF) is a group of proteins containing ubiquitin regulatory X (UBX) domains. According to sequence similarity outside the UBX domain, these proteins are categorised as members of evolutionarily conserved subfamilies [3].

In oncology, several publications have explored the prospective role of Fas-associated factor 1 (FAF1) [4], UBXD proteins [5], and valosin-containing protein (VCP)/p97 [6–11]. This paper, unlike conventional reviews, combines evidence from the scientific literature with high-throughput, multi-omics database evidence to link bioinformatics with experiments to provide a multi-omics perspective on the role of UBXDF in cancers, review the evidence and mechanism of UBXDF’s effects on cancers, and highlight future research opportunities [12].

Bioinformatics evidence was obtained from public databases: Structures of protein domains were collected from cBioPortal [13] and gepia2 [14]. The Cancer Genome Atlas (TCGA) clinical and transcriptome rawdata were collected from UCSC [15, 16]. The complete terms and abbreviations of TCGA cancer types are listed in Additional file 1: Table S1. Staining and protein expression levels of the UBXDF in tumour cells were collected from the Human Protein Atlas (HPA) [17]. Immune cell infiltration was estimated by ImmuCellAI [18] and GSCA [19] with TCGA data. The GSCA [19] and CellMiner [20] were utilised to assess the impact of UBXDF on tumour drug sensitivity. Additional file 1 offer comprehensive data and methods. To compile the literature evidence, English-language PubMed articles published before November 2022 were gathered using keywords (*(“UBXD” OR “UBXN6” OR “UBXN4” OR “UBXN10” OR “UBXN2A” OR “UBXN11” OR “UBXN8” OR “UBXN7” OR “FAF2” OR “ASPSCR1” OR “NSFL1C” OR “UBXN2B” OR “FAF1” OR “UBXN1”) AND (“Cancer” OR “Tumour”)*). To review patterns, the preclinical in vitro, in vivo, and clinical of UBXDF impacts on tumours, in addition to UBXDF molecular mechanism and UBXD tumour drugs, were outlined. In this paper, the UBXD protein and its roles and related mechanisms in cancer are reviewed systematically. The application of the UBXD protein in cancer is thoroughly interpreted, providing new ideas and directions for antitumour drug targets.

## Expression of UBXD family in pan-cancer

### Protein domain structures of UBXD

**Fig. 1:**
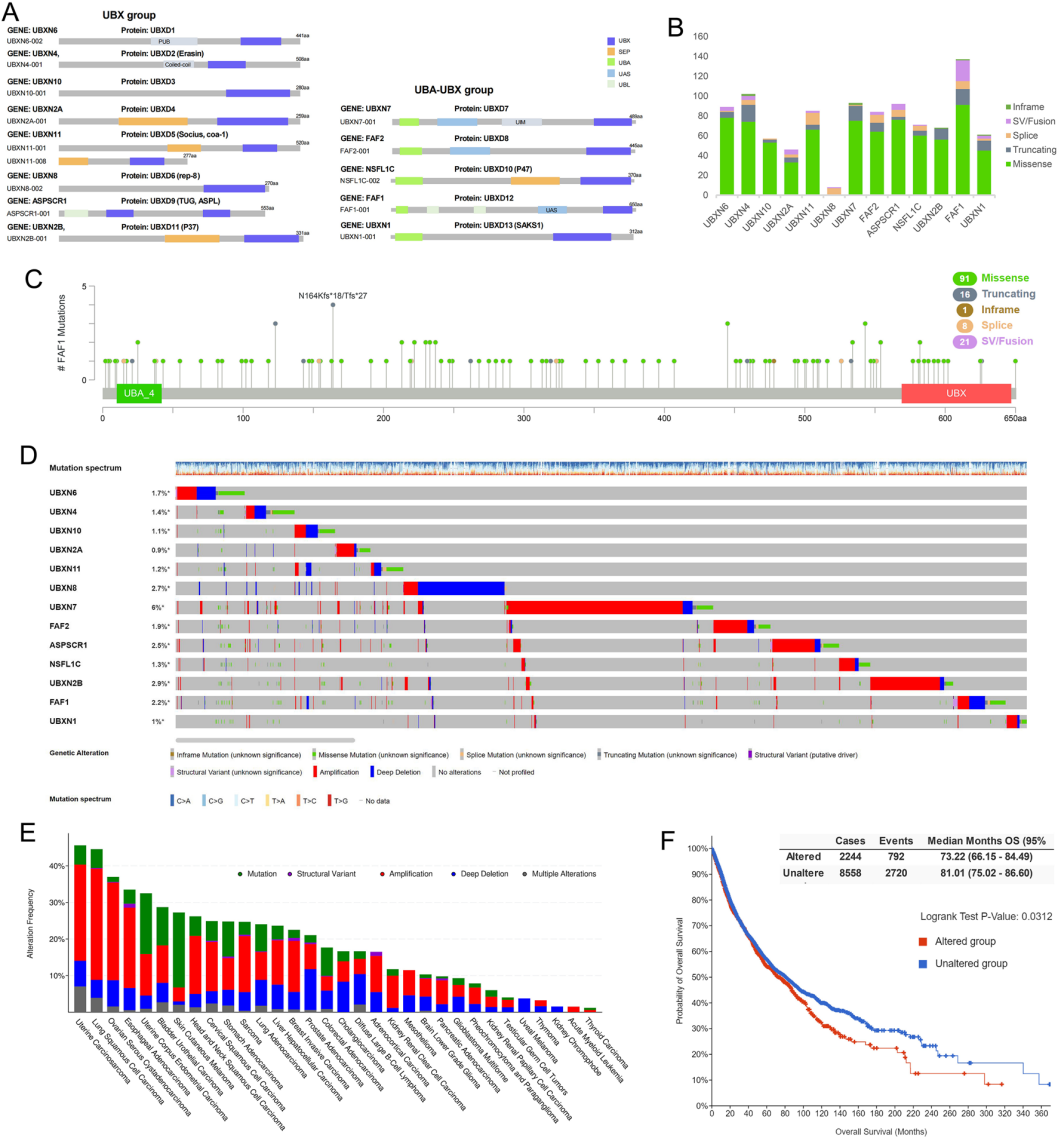
Structure and gene alteration of UBXDF **A** UBXD proteins domain structures and groups. **B** Bar plot of counts of the four mutations for each UBXD gene. **C** The *FAF1* gene mutation locations and count of all TCGA pan-cancer rawdata. **D** The OncoPrint with mutation spectrum and UBXDF gene alteration. **E** The UBXDF mutations frequency in TCGA. **F** K-M plot of the OS (UBXDF altered group vs. UBXDF unaltered group)

Mammalian cells contain 13 members of the UBXDF, which are separated by order of their ubiquitin-related protein motifs: 8 members in the UBX (ubiquitin regulatory X) group and 5 members in the UBA (ubiquitin-associated)-UBX group (Fig. 1A) [21]. In the UBX group, the UBX domain was the only ubiquitin-related domain [22]. UBXD9 is different from other UBXDF members in the UBX group because it has two UBX domains [23]. In addition to the UBX domain, the N-terminal of the protein contains another domain [24, 25]. In the UBA-UBX group, the UBA domain is found at the N-terminal of members, whereas the UBX domain is located at the C-terminal. Some members of UBA-UBX group have additional ubiquitin-related domains like UIM (ubiquitin-interacting motif) and UBL (ubiquitin-like) [22].

Trimerisation of p47 (UBXD10) occurs at its central SEP (Shp1, eyes-closed, p47) domain, and the UBX domain defines the p47 subfamily at its C-terminal. The authentic p47, which has the UBA domain at its N-terminal, belongs to this subfamily [26, 27]. In addition, the relatively distant socius protein has homology with other p47 members in UBX and SEP domains. P47 often acts as an adapter in the homotypic membrane fusion of AAA ATPase (ATPases associated with various cellular activities) p97/VCP. The UBX domain of p47 works directly with p97/VCP, thus mimicking the ubiquitinated substrate. A large part of the function of other family proteins is unknown. Although the UBX contains relevant protein p47 has been discussed as binding to the AAA ATPase Cell Division Cycle 48 (CDC48)/p97 [28]. Recently, scientists realised that the UBX protein is usually a cofactor of p97 and that there was a second p97 binding at the C-terminus of the SEP domain [29]. The FAF1 (UBXN3A) subfamily is distinguished by the presence of a thiodoredoprotein-like folding motif shared by the N-terminal UBA domain, the C-terminal UBX domain, and the central UAS (Upstream activating sequence) domain of unknown function [30]. The true FAF1 homologues are restricted to insects and vertebrates, while the ETEA (UBXD8) and UBXD7 are found in yeast and humans, respectively. The true FAF1 homologue is distinguished by one or two transmembrane domains close to the N-terminus, while the ETEA homologue is characterised by two biguanide-like domains with unknown functions [31]. The true SAKS1 (UBXD13) homologous and erasin-like proteins make up the other half of the SAKS1 subfamily; these proteins are conserved from yeast to humans and have a highly similar central region that is not present in the other UBX subfamilies. SAKS1 contains both an N-terminal UBA and C-terminal UBX domains [32]. Members of the TUG (UBXD9) subfamily can be found in all known eukaryotic organisms, and their central UBX domains reflect this diversity. An intriguing feature of this family is the presence of N-terminal ubiquitin-like domains in some of its members [33]. There is extensive sequence conservation among the UBXD1 subfamily members discovered in all eukaryotes apart from fungi [34, 35]. It has a carboxy-terminal UBX domain and a central PUB (PNGase/UBA or UBX) domain. The N-terminal transmembrane span and C-terminal UBX domain are features unique to the rep8 (UBXD6) subfamily found in vertebrates [36]. And lastly, UBXD3 homologues are observed only in mammals [37].

Due to the presence of a wide variety of domains and their potential permutations within the UBXDF, the individual proteins within this family display a wide range of functional characteristics. These variations enable them to crosstalk with various protein complexes and bind to a limited number of partners that depend on their subcellular localisation [23, 38–41]. Due to the presence of additional ubiquitin-related motifs in addition to the UBX domain, UBXD does not participate redundantly in the ubiquitin-proteasome pathway [42, 43].

### The mutation of UBXD family in tumours

We analysed the UBXDF gene mutations in TCGA. 993 gene mutations of UBXDF in tumours are dispersed throughout the entire protein instead of clustered in the selected locations (Additional file 1: Figure S1A, Fig. 1B). FAF1 has the most gene alterations in the UBXD family (Fig. 1C), and its germline mutations have been linked to hereditary colon cancer [44]. The majority of these regions had a single modification. In contrast, 109 locations had two to eight modifications, indicating that the mutations could result from random mutations accumulated during gene replication. Most UBXDF gene mutations are missense. High levels of gene amplification were found in UBXN7, while deep deletions were found in UBXN8 (Additional file 1: Figure S1B, Fig. 1D). The UBXDF gene was most amplified in LUSC and most deeply deleted in PRAD (Fig. 1E).

The UBX domain contains 80 residues, often located at the C-terminal of eukaryotic proteins. Based on the sequence alignment of structures, proteins containing the UBX domain have been identified in all eukaryotic species [45]. As UBXDF is an evolutionarily conserved subfamily [3] and short proteins subfamily, its overall mutation rate is typically lower in TCGA tumours. UBXN7 (6%) was the only UBXDF gene with a frequency > 3% in the TCGA database. There was a statistically significant difference between overall survival (OS) with and without UBXDF gene mutation (Fig. 1F); UBXDF gene alteration causes poor prognosis (*p* < 0.05), indicating these gene mutations may be associated with the progress of tumour.

### The mRNA expression of UBXD family in tumours

**Fig. 2:**
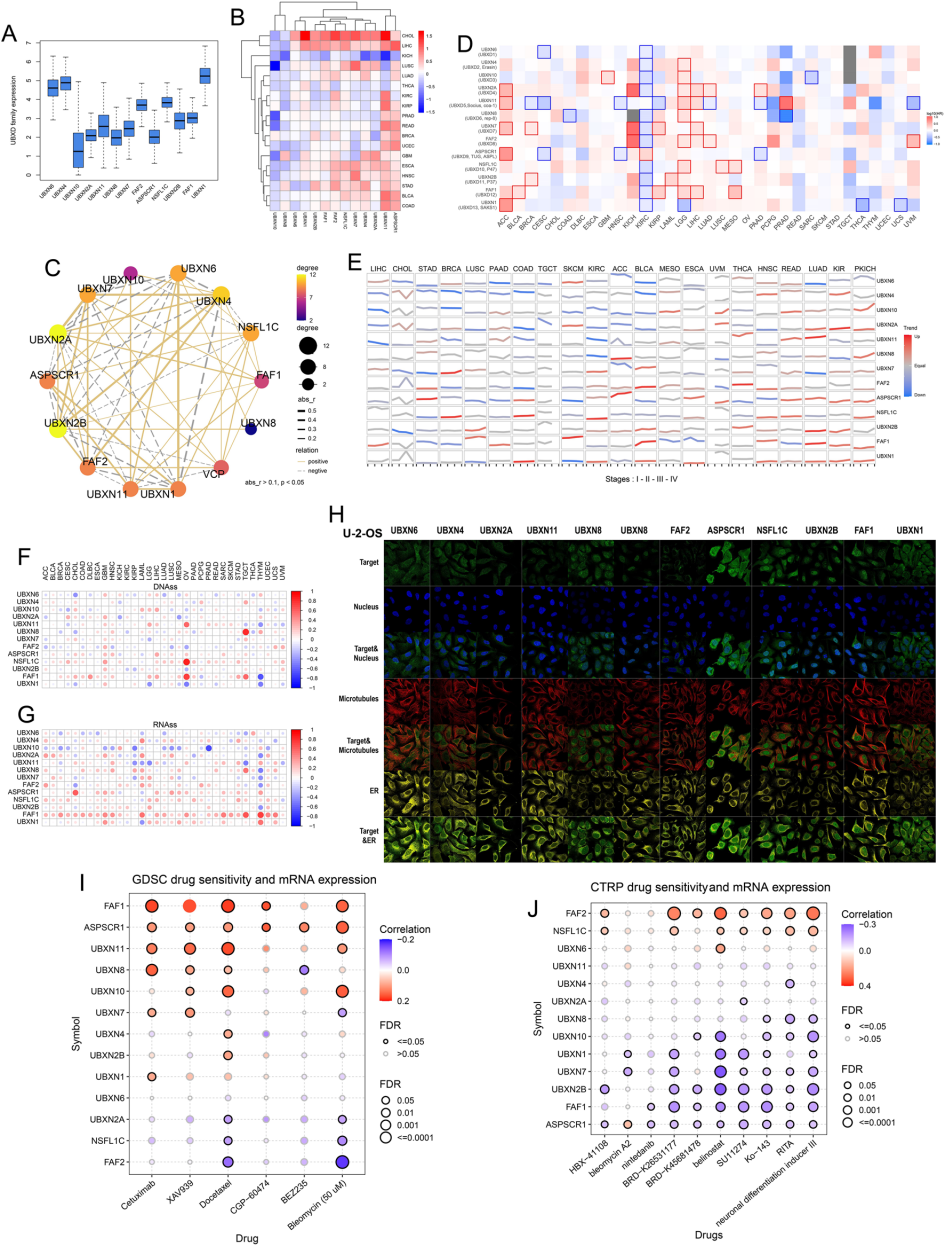
Expression of UBXDF in cancers. **A** The boxplot shows the mRNA expression levels of UBXDF based on the TCGA dataset. **B** Heatmap shows differential UBXDF expression (normal vs. tumour) in TCGA. **C** Co-expression network among UBXDF and *VCP* (p97). **D** Survival contribution of UBXDF genes in 33 cancer types. The coloured border represents a significance level of less than 0.05. **E** Trend plot presents the trend of gene expression from stage I to stage IV. **F**, **G** Correlations between UBXDF expression and DNAss or RNAss. **H** Representative images of UBXDF (except UBXN8) protein in the U-2-OS cell line. **I**, **J** Correlations between UBXDF and drug sensitivity data from the GDSC or CTRP.

To reduce methodological differences between datasets, we analysed the mRNA expression of UBXDF in the TCGA tumours tissue database alone. We observed the expression levels of individual UBXD genes (Fig. 2A). Within the UBXDF, UBXN1 expression was the highest in tumours. To summarise the UBXDF gene expression in various tumour types in TCGA, we constructed UBXDF expression profiles of cancer-noncancerous tissues; however, the sample size (noncancer tissue sample size > = 5) limits the quality of the findings (Fig. 2B, Additional file 1: Table S2A, Figure S2A). We conducted Wilcox test analyses to calculate the significance. Surprisingly, UBXN10 expression was downregulated in the majority of tumours except for KIRP and BRCA, while ASPSCR1 expression was upregulated in tumours except for KICH, KIRC, and THCA. In addition, all UBXDF members were downregulated in KICH. Most members of UBXDF were upregulated in LIHC (*p* < 0.01), except for UBXN10 and UBXN8 (the two members with the lowest expression levels in the major tumours).

### The co-expression of UBXD family in tumours

Protein p97/VCP is a highly conservative type II AAA protein containing two AAA ATPase domains [46, 47]. Many known p97 adapters utilise a conservative binding motif, with the UBX domain being the most common motif [48]. Through the N-terminal domain of p97, the UBX domain allows all groups of the UBXD family (UBXDF) to connect to the multifunction AAA ATPase p97/VCP protein [48–50]. The UBX domain binds to the hydrophobic sac between the two subdomains of the p97 N-terminal domain. Interactions between proteins have shown that p97 is more likely to bind to the UBX domain than ubiquitin [51]. UBXD protein binds to endoplasmic reticulum lumen via p97 and becomes a key cofactor of endoplasmic reticulum-related degradation (ERAD) pathway [52].

We observed significant co-expression among most UBXDF members (*p* < 0.001) (Additional file 1 : Figure S2B, Table S2B), and p97/VCP was significantly co-expressed with all family members except UBXN1 (Fig. 2C). The results were consistent with previous studies; thirteen mammalian proteins have been found to bind to p97 and have been shown to contain a UBX domain [22]. UBXD regulates p97 through the massive interaction networks they create and the structural constraints they impose on p97 as well as its compounds [53]. The correlation network revealed a significant positive co-expression relationship (*r* = 0.58) between UBXN6 and UBXN1. This correlation could be indicated between UBXN7 and UBXN2A (*r* = 0.56) as well. In contrast, there was a negative co-expression among UBXN4 and UBXN1 (*r* = − 0.44).

### The protein expressions of UBXD family in cancers

We used the HPA database to retrieve UBXDF tissue staining results to evaluate protein level expression. In healthy tissue, UBXN4 was highly expressed in most tissues, while UBXN6 was lowly expressed in most tissues, and ASPSCR1 had not highly expressed in the normal tissues. UBXD members are moderately expressed in most normal tissues but are often lowly expressed in muscles (smooth, skeletal, heart) (Additional file 1: Figure S2E). We also observed protein expression in tumour tissues (Additional file 1: Figure S2F).

According to the tissue staining data of UBXD family, we can know that cancer cells show varying degrees of cytoplasmic or nuclear immune reactivity in the UBXD family of proteins. For example, most cancer tissues exhibit weak to moderate cytoplasmic and/or nuclear immunoreactivity in the ASPSCR1, UBXN2A, UBXN10, UBXN1, FAF1, FAF2, UBXD2B proteins. Of UBXN4, UBXN8, and NSFL1C proteins, most malignant cells showed moderate to strong cytoplasmic immune reactivity. Cancer cells exhibit moderate cytoplasmic and/or nuclear immunoreactivity in the UBXN6 protein. The staining results in testicular neoplasms, urothelial, gastric, and pancreatic cancers, and occasional melanoma, breast, and prostate cancers are strongly positive. Some hepatocellular carcinoma, endometrial carcinoma, and kidney cancer showed weak positive or negative. In the UBXN11 protein, cancer cells are weakly stained or negative in most cases, and only a small number of breast, prostate, and pancreatic cancer cases were moderately stained. But breast cancer shows a strong immune response in the subpopulation of cells. Most tumour tissues showed moderate nuclear positivity in the UBXN7 protein, and a small number of skin cancers and rare tumours of the ovary, cervix, lung, and testicle are strongly positive. Additional cytoplasmic positivity has been observed in cervical, endometrial, testicular, liver, and prostate cancers.

Nevertheless, protein expressions were discordant with mRNA expressions for most tumour types or were unavailable. ​The HPA has examples of UBXDF proteins staining in the U-2-OS cell line, where the majority of UBXDF members are found and are predominantly located in the nucleoplasm (UBXN7, ASPSCR1, NSFL1C, UBXN2B, FAF1, UBXN1, and UBXN8) (Fig. 2H).

## Role of UBXD family in pan-cancer prognosis

### UBXD family and prognosis of cancer patients

To assess the prognostic cancer value of UBXDF mRNA expression, we constructed 68 K-M curve plots of overall survival across cancer types and UBXDF members, with *p* < 0.05 (Additional file 1 Figure S2C). The expression level of some UBXDF members is significantly correlated with cancer patients’ overall survival and may be involved in cancer progression (Fig. 2D). In KIRC, 11 genes in the UBXDF were significantly negatively correlated with OS, and 1 gene (ASPSCR1) was significantly positively correlated with OS. In LGG, eight genes in the UBXDF were significantly positively associated with OS, and 1 gene (UBXN1) was significantly negatively associated with OS. These findings indicate that the UBXD gene family is closely associated with cancer and may serve as a biomarker and prognostic indicator for various cancers. Finally, low expression of UBXDF genes (except for UBXN4, UBXN10, and UBXN2A) in DLBC causes poor prognosis, whereas high expression of UBXDF genes (except for UBXN6) in KICH causes poor prognosis (Additional file 1: Figure S2D, Table S2C). In the analysis of mRNA expression trends and stages (Fig. 2E), we found that the expression trends of UBXDF were significantly correlated with most cancers. Most UBXDF expression trends increased with the increase of stage in KICH, and UBXDF could play a crucial role in the development of KICH stage.

Additionally, we analysed the UBXDF correlation of cancer stemness, which was strongly correlated with cancer prognosis. Stemness represents the loss of a differentiated trait and the acquisition of progenitor and stem-cell-like properties [54]. For DNA methylation-based stemness index (DNAss), that was notable to find a strong positive association between OV and NSFL1C (*r* = 0.85, *p* = 0.023) as well as TCGT and UBXN8 (*r* = 0.80, *p* < 0.001). FAF1 has a strong negative correlation with THYM (*r* = − 0.65, *p* < 0.001) (Fig. 2F, Additional file 1: Table S2D, E). For mRNA expression-based stemness index (RNAss), UBXN10 has a strong negative correlation with PRAD (*r* = − 0.69, *p* < 0.001). FAF1 has a strong positive correlation with THCA (*r* = 0.74, *p* < 0.001) (Fig. 2G, Additional file 1: Table S2F, G). The results revealed that some members of UBXDF were strongly correlated with stemness. As a result, we concluded that FAF1 might be an effective prognostic biomarker for those tumours. According to our review, no studies have investigated the cancer stemness of FAF1. Additionally, the stemness of UBXDF in tumours has received little research [55]. It is uncertain whether stemness of UBXDF can guide cancer prognosis. We believe that studies between UBXDF and cancer stemness are urgently needed.

### UBXD family and cancer tumour drugtherapy

To explore the effect of UBXDF on drugtherapy, we analysed associations of UBXDF transcriptome levels with therapeutic responses (drug sensitivity) (Additional file 1: Figure S2F). ​We found a negative link between UBXN8 expression and drug sensitivity such as everolimus, AP − 26113, and denileukin diftitox ontak, between NSFL1C and drug sensitivity such as dolastatin 10, vinblastine, and vinorelbine. At the same time, UBXN1 expression was positively correlated with cladribine, 5-fluoro-deoxy uridine 10mer, and fludarabine. The expression of FAF1 was negatively correlated with alectinib.

Furthermore, we analysed the relationship between UBXDF expression and the sensitivity (IC50) of cancer cell lines to various drugs using datasets from the GDSC [56] and CTRP [57], which include detailed info on cancer cell lines. Remarkably, half of UBXDF member expressions were inversely linked with the IC50 of cancer cell lines in CTRP, among these significant connections (Fig. 2J, Table 2H), while positively correlated in GDSC (Fig. 2I, Table 2I). These findings suggest that UBXDF may be a valuable predictive biomarker for pharmacological therapy; however, further research is required.

### UBXD family and tumour microenvironment

Immune molecules and immune cells inside the tumour microenvironment (TME) are essential variables influencing carcinogenesis and can determine the responsiveness of malignancies to immunotherapy [18]. Consequently, it may be helpful to examine the relationship between UBXDF and immunological molecules to investigate the possible influence of UBXDF on TME and immunotherapy.

Solid tumour tissue includes tumour cells and immunological, stromal, and vascular cells. We used ESTIMATE [58] to calculate tumour stroma, purity, and immune score in TCGA, and the spearman correlation between UBXDF expression and scores were evaluated (Additional file 1: Table S3A, Figure S3A–D). FAF1 expression was inversely correlated with SARC, TGCT, THYM, and UCS, indicating that elevated FAF1 expression may be associated with reduced tumour purity. The role of FAF2 between ACC was the same as FAF1, and it was negatively associated with the stromal and immune score but positively associated with tumour purity.

**Fig. 3:**
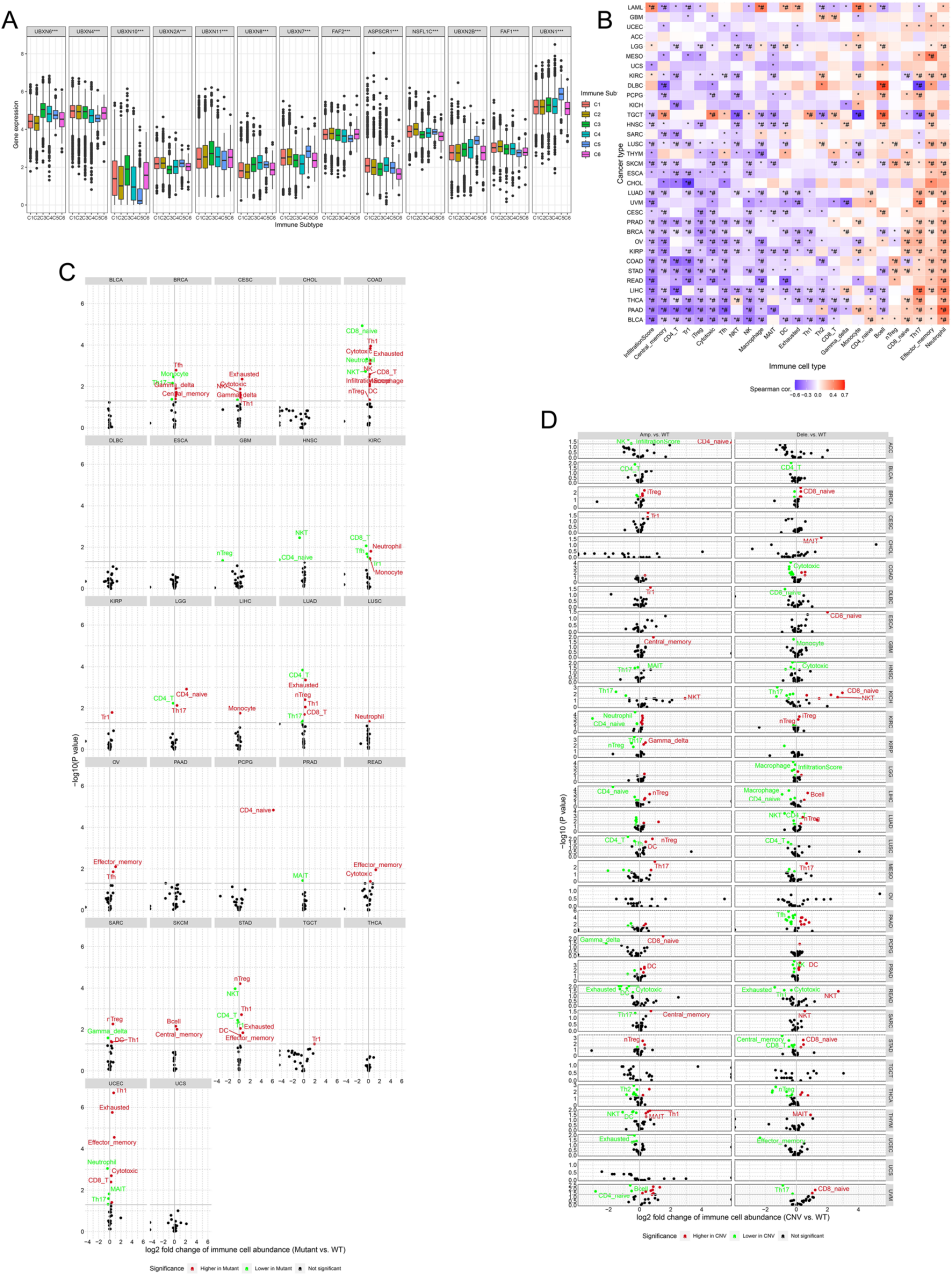
UBXDF and cancer immunity. **A** Immunological subtype analysis of UBXDF genes in TCGA tumours. (∗∗∗*p* < 0.001) **B** Heatmap summarises the significance of *p*-value and FDR for the spearman correlation analysis between GSVA score of the inputted gene set and immune cells’ infiltrates. (*p ≤ 0.05; #*FDR* ≤ 0.05) **C** The scatter plot summarises the significance of *p*-value and *FDR* for comparing mean infiltrate between SNV groups. Using colour to indicate the *p*-value significant (green) and *FDR* significant (red) results. **D** The scatter plot summarises the significance of *p*-value and *FDR* in comparing mean infiltrate between CNV groups. Using colour to indicate the *p*-value significant (green) and *FDR* significant (red) results

The five primary hallmarks of tumour immune-expression are macrophages/monocytes [59], total lymphocyte infiltration (mainly T and B cells) [60], TGF-β response [61], IFN-γ response [62], and wound healing [63]. Based on the aforementioned immune-expression patterns, it is possible to classify all cases into six repeatable immunological subgroups [64]. Using the Kruskal-Wallis test, we investigated UBXDF gene expression in six immunological subgroups of TCGA pan-cancer. Statistically, the expression levels of all thirteen UBXDF members were distinguishable amongst immunological subgroups (Fig. 3A) that are not limited to specific tumour types and may play a crucial role in prognosis prediction [64].

Finally, to build a comprehensive profile of UBXDF in immunity across cancer, we determined the relationship between immune cell infiltration and UBXDF gene set expression level (GSVA score) [65], single nucleotide variant (SNV) level, and copy number variation (CNV) level. The infiltrates of 24 immune cells were evaluated through ImmuCellAI [18]. Strikingly, UBXDF expression level was negatively correlated with infiltration score, central memory, CD4 T, Tr1, Treg, cytotoxic, Tfh, NKT, NK, Macrophage, and MAIT. On the other hand, UBXDF was positively correlated with neutrophil and effector memory in most cancer types (Fig. 3B). The gene set SNV level represents the integrated SNV status of inputted gene set for each sample. Immune-cell infiltration differed significantly between mutant and wild-type mutation groups in most cancers (Fig. 3C). The gene set CNV level represents the integrated CNV status of inputted gene set for each sample. We noted that Th17 immune infiltration was significantly downregulated, and NKT immune infiltration was significantly upregulated in the amplified and deleted groups for the WT group in KICH (Fig. 3D). These findings revealed the biomarker potential of UBXDF in immunotherapy targeting NKT, neutrophil, or effector memory cells. In addition, the data found a correlation between UBXDF and Th17 in certain tumours, indicating that it may influence IL-17 release in these tumours.

A significant disadvantage of these correlation analyses is that a troubling connection may occur in certain forms of cancer due to the small sample size. Consequently, we must exercise caution when interpreting this data. More research is necessary to investigate the potential immunotherapy impact of UBXDF.

## Literature evidence for UBXD family involvement in cancer

### In vitro evidence of UBXD family involvement in cancer

Numerous articles have presented in vitro evidence for the role of UBXDF in cancer (Table 1). It has been reported that some members of the UBXDF play a key role in the dysregulated formation, growth, proliferation, migration, invasion, and apoptosis pathways in specific tumours.

**Table 1:** In vitro evidence for roles of UBXDF in cancers

Protein	Cancer type	cell line	Effects	Mechanisms	References
UBXN10	Colon adenocarcinoma	FHC, HCT116, SW480 and SW620	Inhibits proliferation and migration	Modulates the miR-515-5p/SLIT3 axis	[66]
UBXN2A	Colorectal cancer	HCT-116, LoVo, MCF7, U2OS, HeLa and HepG2	Promotes apoptosis, inhibits proliferation	Regulates the protein level of mot-2	[67]
UBXN2A	Colorectal cancer	HCT116, LoVo, SW-480 and U2OS	Promotes cell death	Regulates the oncoprotein mot-2	[68]
UBXN2A	Colorectal cancer	HCT-116 and LoVo	Promotes apoptosis	Regulates the oncoprotein mot-2	[39]
UBXN2A	Colorectal cancer	HEK293, HeLa, HCT-116, MCF7, SW48, HT-29, SW620, T84 and HUVEC	Promotes apoptosis, inhibits cell growth	A p53-dependent pathway	[69]
UBXN2A	Osteosarcoma	HEK-293T, HCT-116, LoVo and U2OS	Promotes apoptosis, inhibits proliferation and migration	The mortalin oncoprotein pathway	[70]
UBXN8	Hepatocellular carcinoma	HepG2, Hep3B, HuH1, HuH7, SMMC7721, SNU182, SNU387, SNU449, PLC/PRF/5 and Sk-Hep-1	Inhibits cell growth	Regulates the expression of cell cycle negative regulators TP53 and p21CIP1/WAF1	[71]
UBXN7	Hepatocellular carcinoma	HEK293, Huh7 and HepG2	Inhibits autophagy	NF-κB signalling pathway	[72]
UBXN7	Lung squamous cell carcinomas	H520, HCC95, H2882, HCC15, H157 and SW900	Promotes cell growth	Identification of candidate drivers in Chromosome 3q26-29	[73]
FAF1	Breast cancer	/	Inhibits invasion and metastasis	TGF-β signalling pathway	[74]
FAF1	Breast cancer	HEK293T, HeLa, MCF7, MDA-MB-231 and MDA-MB-435	Promotes cell growth	TGF-β signalling pathway	[75]
FAF1	Breast cancer	HEK293, HeLa, MCF7 and MDA-MB-23	Inhibits migration	Wnt/β-catenin signalling pathway	[76]
FAF1	Colorectal cancer	DLD-1	Promotes apoptosis	NF-κB and Wnt signalling pathway	[44]
FAF1	Cervical cancer	HEK293T and HeLa	Inhibits tumor formation	Regulates the degradation of Hsp70	[77]
FAF1	Clear cell renal cell carcinoma	SRD-13 A and HEK-293	Inhibits cell growth	Regulates the degradation of β-catenin	[78]
FAF1	Lung cancer	A549 and H1299	Inhibits cell growth, migration and invasion, promotes apoptosis	Wnt signalling pathway	[79]
FAF1	Gastric cancer	HGC-27	Promotes cell death and apoptosis	Primarily biochemical pathways	[80]
FAF1	Gastric cancer	HGC-27	Inhibits proliferation and growth, promotes apoptosis	NF-κB signalling pathway	[81]
FAF1	Gastric cancer	BOSC23 and HeLa	Promotes apoptosis	NF-κB signalling pathway	[82]
FAF1	Non-small cell lung carcinomas	A549, H460, H1299, H1650 and SPC-A1	Inhibits proliferation, invasion and migration, promotes cell cycle arrest and apoptosis	TGF-β signalling	[83]
FAF1	Ovarian cancer	/	Negative correlation with HSP70 expression	NF-κB and ubiquitin proteasomal pathways	[84]
FAF1	Prostate cancer	293T, HeLa, HGC-27, MGC-803 and DU-145	Promotes apoptosis	Correlates with the amount of miR-24	[85]
UBXN1	BRCA1 tumour	293 cells	Inhibits the enzymatic function of BRCA1	BRCA1 pathway	[86]
UBXN1	Colon adenocarcinoma	SW480 and HT29	Inhibits cell growth and tumorigenesis	Regulates IκBα levels and the nuclear levels of NF-κB and p-NF-κB	[87]
UBXN1	Diffuse gliomas	H4, LN229 and U87	Inhibits proliferation and migration	NF-κB signalling pathway	[88]
UBXN1	Glioblastoma	N33 and U87-MG	Inhibits cell growth and tumorigenesis	NF-κB signalling pathway	[87]
UBXN1	Glioblastoma	U87 and LN229	Inhibits cell growth	Regulates the level of NF-κB	[89]
UBXN1	Prostate cancer	PC3, LNCaP, 22Rv1 and DU145	Promotes proliferation, migration and invasiveness	PI3K/Akt/NF-κB Pathway	[90]

UBXN10-AS1 [66] showed a trend of low expression in colon adenocarcinoma tissues. UBXN10-AS1 acts as a tumour suppressor, regulating the miR-515-5p/SLIT3 axis, and the overexpression of UBXN10-AS1 could inhibit the proliferation of COAD cells in vitro and in vivo, working as an antitumour role. UBXN2A binding to the starch-binding domain of mot-2 can control or limit the development of colorectal tumour cells by competitively disrupting the p53-mot-2 connection [39, 67–69]. UBXN8 is an endoplasmic reticulum transmembrane protein that binds p97 to misfolded ERAD proteins. Low expression of UBXN8 interferes with this process, causing misfolded or unassembled proteins to accumulate in the endoplasmic reticulum lumen, which in turn induces endoplasmic reticulum stress. Endoplasmic reticulum stress can induce cytoplasmic localisation and degradation of p53. Therefore, UBXN8 can regulate the expression of the cell cycle inhibitors TP53 and p21CIP1/WAF, which function as tumour suppressors in hepatocellular carcinoma [71]. As an adaptor protein of CRL2/VHL ligase complex and a specific substrate of MUL1 ligase, UBXN7 regulates HIF-1α protein expression under aerobic or anaerobic environments. The interaction between UBXN7 and cullins is not mediated by its ubiquitinated substrate but involves the UIM motif in UBXN7 directly with the ubiquitinated cullins [40]. UBXN1, p47, and FAF1 can target and inhibit crucial proteins involved in tumourigenesis as well as development and block the transcription of oncogenes activated by NF-κβ pathway. UBXN1 can regulate Iκβα expression and nuclear expression of NF-κβ and p-NF-κβ to control the development and tumourigenesis of cancerous cells. The physical interaction of FAF1 with IKKβ disrupts the assembly of IKK complexes, inhibiting NF-κβ activity and its downstream signalling pathways [4]. FAF1 renders TβRII unstable at the cell surface by recruiting the VCP/E3 ligase complex, thereby avoiding an excessive TGF-β response [75]. Markedly activated AKT directly phosphorylates FAF1, destroying the FAF1-VCP complex and reducing FAF1 on the plasma membrane. The latter promotes TGF-β-induced SMAD & non-SMAD signals and increases TβRII expression on the cell surface.

### Preclinical in vivo evidence of UBXD family involvement in cancer

The role of UBXDF in cancers has also been verified in xenograft mouse models (Table 2). In colon adenocarcinoma [90], UBXN10-AS1 is expressed at low levels and is predominantly localised in the cytoplasmic portion of COAD cells. Overexpression of UBXN10-AS1 reduced the proliferation and migration of COAD cells in vitro and slowed down the growth of tumours in vivo. Protein UBXN2A containing UBX domain can promote ubiquitination and proteasome degradation of mot-2 protein mediated by ubiquitin E3 ligase CHIP. The level of UBXN2A protein in colon tumour tissues is markedly lower than that in adjacent normal tissues. Enhancement of UBXN2A leads to apoptosis at the cellular level and in living animals, thereby inhibiting tumour growth, reproduction, and metastasis [67–69]. TGF-β can promote the metastasis of advanced breast cancer cells. TβRII accumulated in FAF1-deficient cells of mouse embryos in FAF1-knockout mice, indicating that FAF1 has the physiological function of inhibiting TβRII [75]. In Non-small cell lung carcinomas [83], Sanguinarine can increase the expression of FAF1. The up-regulated FAF1 inhibits cell proliferation, invasion, and migration and induces cell cycle arrest and apoptosis. This finding confirms that FAF1 can serve as a new therapeutic target. Studies on the progression of tumours in asbestos-induced malignant mesothelioma mouse models have shown that FAF1 is an essential factor in regulating the NF-β pathway. As in mouse model, the loss of FAF1 may relate to aberrant NF-β signalling and tumour progression [91]. The expression of YTHDF2 in diffuse gliomas [88] promotes the deterioration of gliomas, and UBXN1, as a protein containing the UBX domain, inhibits the activation of NF-κβ by maintaining the expression of Iκβα. Its expression can inhibit glioma cell proliferation and migration stimulated by YTHDF2 upregulation. In Glioblastoma and Colon adenocarcinoma [87], FAF1 inhibits cell growth and carcinogenesis through TNF-triggered NF-β signalling.

**Table 2:** Preclinical in vivo evidence of UBXDF effect on cancers

protein	Cancer type	Samples	Effects	References
UBXN10	Colon adenocarcinoma	Xenograft mouse model	Inhibits tumour growth	[66]
UBXN2A	Colorectal cancer	Xenograft mouse model	Inhibits proliferation and migration	[67]
UBXN2A	Colorectal cancer	Xenograft mouse model	Inhibits tumour growth	[68]
UBXN2A	Colorectal cancer	Xenograft mouse model	Inhibits tumour growth	[69]
FAF1	Breast cancer	Xenograft mouse model	Inhibits metastasis	[75]
FAF1	Non-small cell lung carcinomas	Xenograft mouse model	Reduces tumour volume and weight	[83]
FAF1	Malignant mesothelioma	Xenograft mouse model	Inhibits MM development	[91]
UBXN1	Diffuse gliomas	Xenograft mouse model	Inhibits tumour progression	[88]
UBXN1	Glioblastoma	Xenograft mouse model	Inhibits tumour growth and tumourigenesis	[87]
UBXN1	Colon adenocarcinoma	Xenograft mouse model	Inhibits tumour growth and tumourigenesis	[87]

### Clinical evidence

FAF1 is a tumour suppressor gene that plays a role in various cancers. In a recurrent leiomyosarcoma study [92], analysis of DNA exon sequences, RNA and protein expression, and transcription factor binding in sarcomas and unaffected muscles and bones revealed that the cause of the disease was a point mutation S181G in FAF1, which may lead to loss of apoptotic function following transformed DNA damage. The loss of FAF1 function may affect the activity of the constitutive Wnt pathway and promote the occurrence of leiomyosarcoma. To fully comprehend how UBXDF impacts cancer cells, further studies are needed.

### UBXD family targeting drugs

Adult T-cell leukaemia/lymphoma (ATLL) is a malignant tumour caused by human T-cell leukaemia virus type 1 (HTLV-1) infection. A previous study [93] revealed that chloroquine (CQ) or hydroxychloroquine (HCQ), an FDA-approved antimalarial drug, induced apoptosis and inhibited ATLL cell growth in vitro and in vivo. Autophagy was inhibited in CQ or HCQ-treated ATLL cells, which promoted the recovery of the negative regulator p47 (NSFL1C) and the inhibition of NF-κB activation, triggering ATLL cell apoptosis.

Abdullah et al.‘s significant work [68] demonstrated through high-throughput drug screening that veratridine, a natural plant alkaloid, upregulates UBXN2A expression in cancer cells. This upregulation induces increased cell death and inhibits cell proliferation, especially in colon cancer lines, highlighting the potential of targeting UBXD family proteins in cancer therapy [68].

Additionally, in various cancers, including lung cancer, Abnormal de novo lipid synthesis contributes to the progression and therapeutic resistance of various cancers, including lung cancer. Orlistat (an FDA-approved anti-obesity drug) inhibited tumour growth in human and mouse cancer cells (in vivo and in vitro) [94]. Using RNA-seq to explore changes in genome-wide gene expression profiles mediated by orlistat treatment, FAF2/UBXD8 was found to be a new target associated with lipid metabolism in many significantly affected genes, and knockout of FAF2 further enhanced orlistat-induced survival inhibition, whereas overexpression of FAF2 reversed. Nevertheless, the potential mechanism of orlistat inhibiting FAF2 remains to be further explored.

### Protein partners of UBXD family and their network

The UBXDF family, known for its wide-ranging interactions with various protein partners, significantly influences cellular functions and cancer pathology. Raman et al.‘s thorough research identified 169 interacting proteins (54 unique) of 13 UBXDF members using N- and C-tag anti-FLAG and anti-HA AP–MS studies [37]. Riehl’s team, using GFP-tagged UBXD9 AX2 strains and a new BirA-UBXD9 strain, discovered 185 potential binders to UBXD9, notably including p97, UBXD9, and GSIII, across multiple methods [95].

Each UBXDF member plays a distinct role in various biological and pathological processes. UBXD3‘s involvement in ciliogenesis, particularly its interaction with the intraflagellar transport B (IFT-B) complex, links it to tumorigenesis, presenting a new perspective on cancer development associated with defective ciliogenesis [37, 96–98]. UBXD4 emerges as a potential cancer therapeutic target due to its interactions with E3 ubiquitin ligases and its role in proteasomal degradation pathways, including its modulation of p53 tumour suppressor proteins [52, 70, 99]. Furthermore, UBXD5’s identification as an antigen in colon tumour-reactive T cells by Maccalli et al. positions it as a promising target for immunotherapy in colorectal and melanoma cancers [100, 101]. In the context of hypoxia response, UBXD7’s targeting of HIF-1α for degradation via interactions with the p97 complex and CUL2/VHL E3 ubiquitin ligase complexes open new avenues for targeting hypoxic tumours [22, 40].

UBXD8’s regulation of neurofibromin, influencing the Ras-mediated signalling pathway, is particularly notable. Phan et al.‘s discovery that UBXD8 silencing reduces Ras activity suggests its potential in treating neurofibroma [102]. UBXD9’s involvement in cellular dynamics is also significant, interacting with actin cytoskeletal proteins and implicating it in processes like Golgi reassembly and vesicle redistribution [25, 103–105].

UBXDF, through its diverse protein interactions, plays a critical role in various cellular processes and diseases, particularly cancer.

### The mechanism of UBXD family in cancer

Numerous UBX proteins contain the UBA domain, which displays conservative permutation within the UBX family compared to the UBX domain. FAF1, SAKS1, p47, UBXD7, and UBXD8 have their UBA domains relatively close to their respective N-terminal, suggesting that these proteins can be “linking” regions between the UBA and UBX domains, making it possible to attach additional cofactors and substrates within a smaller volume than p97. Thirteen mammalian proteins have been found to bind to p97 and have been shown to contain a UBX domain [22]. UBXD regulates p97 through the massive interaction networks they create and the structural constraints they impose on p97 as well as its compounds [53]. There may not be sequential conservation in some structural characteristics of UBX proteins concerned with their function in the p97 complex. These characteristics include the tendency toward oligomerisation, the universality of the second binding site for p97, and the protection of various domain configurations. p97 contributes to regulating protein homeostasis, and tumour cells are highly dependent on protein quality control mechanisms, showing that p97 is a potential therapeutic target for cancer. What’s more, the expression level of p97 is upregulated in many cancers, including human melanoma and breast cancer [10, 106–108].

**Fig. 4:**
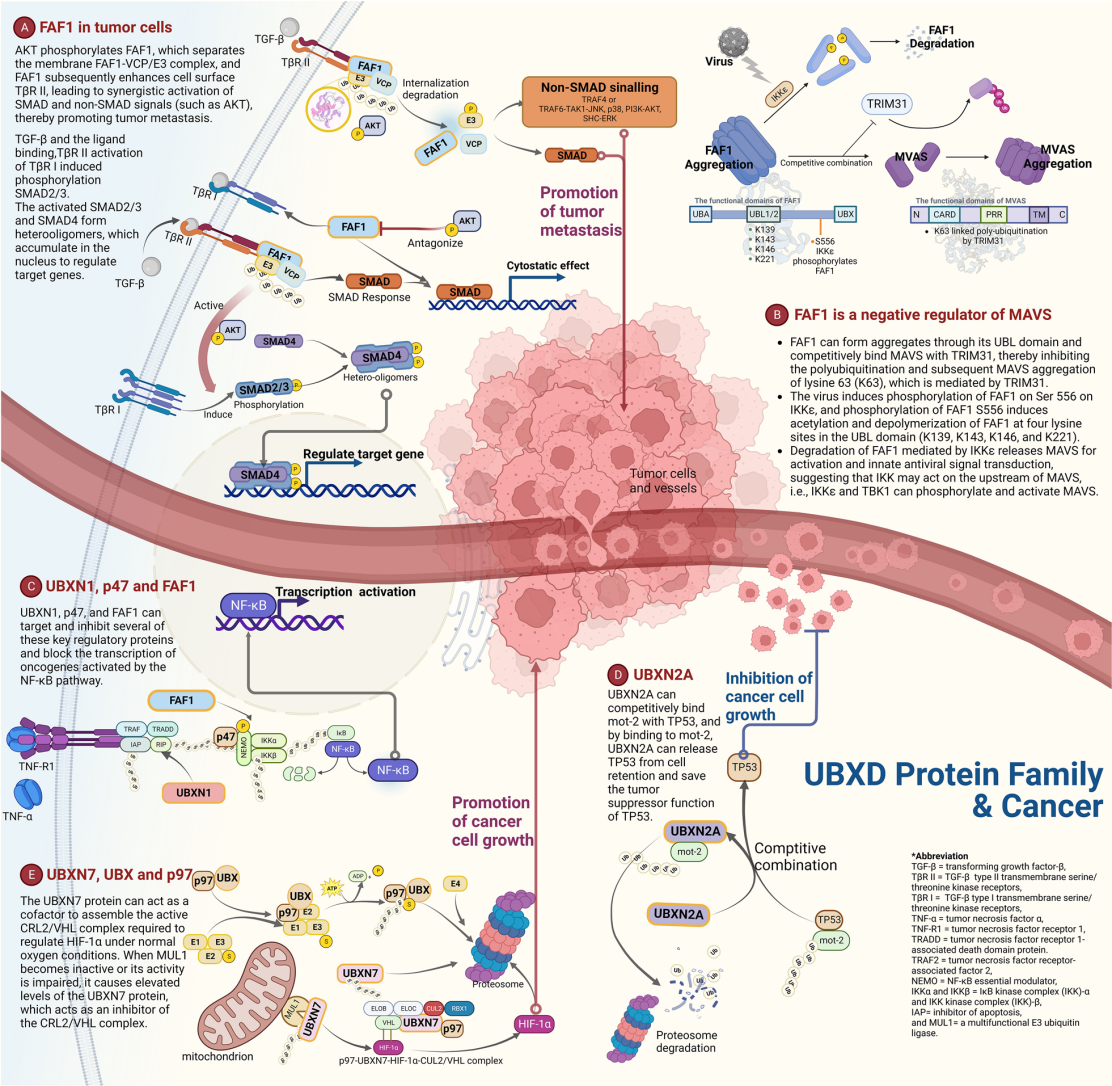
The Mechanism of UBXD family in Cancer. (Created in Biorender.com) Additional file 2

FAF1 is a key regulator of TβRII on the cell surface and prevents overactivation of SMAD and non-SMAD TGF-β-induced signals (Fig. 4A). During cancer development, AKT activation mediates FAF1 phosphorylation and subsequent dissociation of FAF1 from the plasma membrane and TβRII, thereby enhancing the cell surface stability of TβRII and activating TGF-β-induced pre-metastatic function in breast cancer cells [109]. Abnormal AKT overactivation may also alter TGF-β intracellular signalling, thus providing a catalyst for TGF-β’s transformation from tumour suppressor to promoter. Thus, AKT-mediated FAF1 protein inactivation confirmed high expression of cell-surface TβRII, further enhancing SMAD and AKT (one of the non-SMAD pathways) signalling. Thus, AKT, through FAF1 inactivation, triggers a tumour-promoting self-reinforcing cycle of the TGF-β pathway, thereby stimulating cancer cell invasion and metastasis.

It’s also worth noting that FAF1 acts as a negative regulator of mitochondrial antiviral signalling (MAVS) (Fig. 4B). Innate immune receptor retinoid-induced gene 1 (RIG-I) is linked to antiviral signalling through mitochondrial antiviral signalling proteins (MAVs), which mediate the recognition of viral RNA. After interacting with RIG-I, MAVs trigger downstream signalling effectors by polyubiquitinating lysine 63 (K63) with the E3 ligase TRIM31. Inhibiting TRIM31-mediated polyubiquitination of K63 ligation and MAVs aggregation, FAF1 can form aggregates and bind to MAVs via its UBL domain. By acetylating four lysine sites (K139, K143, K146, and K221) in the UPL domain of FAF1, virus-induced phosphorylation of FAF1 at Ser556 promotes FAF1 de-aggregation [110].

UBXN1, p47, and FAF1 can target and inhibit key regulatory proteins in tumourigenesis and development and block the transcription of oncogenes activated by NF-κβ pathway (Fig. 4C).

UBXN2A was originally identified as a function protein controlling the protein transportation of nicotinic receptors in the neural system [99]. Sane et al. [69] reported that in colon cancer cells, UBXN2A could bind to mot-2, inhibiting the binding of mot-2 to p53. Genetic analysis showed that UBXN2A was bound to the substrate binding region of mot-2 and partially overlapped with the p53 binding site, indicating that UBXN2A and p53 may competitively bind to mot-2. UBXN2A protects the tumour suppressor function of p53 by binding mot-2 to release p53 from cellular fixation, which suggests that UBXN2A can promote cell death by interfering with the interaction of p53-mot-2 in colon cancer cells (Fig. 4D).

UBXD7 functions as an adaptor of p97 ATPase, which is essential for the p97-mediated degradation of misfolded or damaged proteins by the ubiquitin-proteasome system (UPS) [40, 111]. When ubiquitinated substrates are present, UBXD7 binds to them via its UBA domain and then recruits p97 or p97 core complexes via its UBX domain’s interaction with the p97 N-terminal domain. Both UBA and UBX domains are inactive due to intramolecular or intermolecular interactions. As a transcription factor, hypoxia-inducing factor 1 (HIF-1) plays an important role in tumours after hypoxia, promoting tumour aggressiveness and possibly damaging the response to radiation and chemotherapy [112]. Reducing HIF-1 levels can disrupt multiple pathways, including cell survival, glucose metabolism, invasion, and angiogenesis [113]. With the UBA domain of UBXD7 binding to ubiquitinated HIF-1, UBXD7 can actively promote the interaction between p97 and CUL2/VHL E3 ubiquitin ligase and HIF-1 PCR. In another study [40], researchers discovered that UBXD7 binds to the neddylated form of CUL2 and uses its UBA and UBX domains to recruit ubiquitinated HIF-1 and p97 complexes. Nevertheless, the excessive expression of UBXD7 showed that the docking mechanism of UBXD7 negatively regulates CUL2 ubiquitin ligase activity and leads to the accumulation of HIF-1. Both two studies have shown that it is complicated the regulation of UBXD7 in the ubiquitin-proteasome pathway (Fig. 4E).

UBXN6 collaborates with protein tyrosine phosphatase 4a2 (PTP4A2) to assemble the endo-lysosomal damage response (ELDR) complex, thereby promoting autophagosome formation and facilitating the clearance of damaged lysosomes [114].UBXN4 exhibits a negative correlation with macrophage-related markers, suggesting its potential as a prognostic marker for lung cancer [115]. Its involvement in regulating the WNT secretory factor EVI/WLS at the protein level further underscores its significance [116]. Meanwhile, UBXD5, encoding the colorectal cancer neoantigen Colon antigen-1 (COA-1), induces peripheral blood mononuclear cell (PBMC) antigen and tumour-specific CD8 + immune responses, highlighting its immunogenic potential [117]. On the epigenetic front, UBXN1 undergoes silencing through promoter region methylation mediated by the RUNX8-RUNX1T1 fusion protein, resulting in significant inhibition of acute myeloid leukaemia (AML) proliferation [118]. ASPSCR1 and TFE3 fusion not only regulates the activity of a super-enhancer (SE) but also promotes Alveolar Soft Part Sarcoma (ASPS) angiogenesis [119].

In summary, these diverse roles of UBXN family members underscore their importance in various cellular processes and disease contexts.

## Conclusions and perspectives

Undeniably, both the bioinformation evidence of TCGA and the evidence in the literature have their limitations. Although TCGA data provides relatively reliable information with a large amount of clinical data and the results of high-throughput mRNA sequencing, it is less customised and cannot provide in-depth investigation. Instead, prospective literature studies can provide well-designed investigations with well-validated evidence if appropriate hypotheses need to be tested. These studies may be affected by limited research resources and biases inherent in the assumptions. Therefore, combining and comparing bioinformatics and literary studies makes sense to obtain a complete view of a discipline. Therefore, we summarise the bioinformatics analysis and literature evidence collected in this paper in Table 3.

Despite understanding the UBXD family action mechanisms in some cancers, current data suggest that UBXD family members play an important role in different types of cancer. Two major areas of UBXDF in cancer that have been less studied are the effect of UBXDF on drug therapy and the impact of UBXDF on immunotherapy. Bioinformatics and literature studies have demonstrated the potential effect of UBXDF on cancer susceptibility to anticancer drugs. The role of UBXDF in drug action may vary depending on the pharmacological mechanisms of the drug, highlighting the value of screening to search for drugs associated with UBXDF. Although several previous studies have examined drugs targeted by UBXDF, this review is the first to summarise potential candidates (Fig. 2I, J). FAF1 inhibits tumour growth, migration, invasion, and apoptosis by regulating signal transduction pathways in breast cancer, stomach, lung, and other tumours. However, little research has been done on drugs targeting FAF1, which could be a breakthrough in developing UBXDF drugs.

From another perspective, UBXDF may be associated with immune scores for several cancer types (Additional file 1: Figure S3D), a conclusion that can be drawn from our bioinformatics analysis results. This type of cancer will be the subject of future research. In addition, 13 UBXDF members showed statistically significant differences in immunological subgroups (*p* < 0.001) [64]. This bioinformatics data suggests that UBXDF may impact pan-cancer immunotherapy (Fig. 3A). Up to now, no research has explored the potential impact of UBXDF on cancer immunotherapy. We believe this is a new direction for UBXDF’s future cancer research.

Colorectal cancer and lung cancer are currently the most studied types of cancer. In future studies, we will study some types of cancer that are less studied but may be potentially affected by UBXDF based on bioinformatics. For example, THYM, whose survival is related to TCGA-based FAF1, has not been studied. We also propose to study further some less concerned members of the UBXD family, which may also affect tumour formation and progression in vitro and in vivo. For example, UBXD5, which encodes the carboxyl-terminal of COA-1, has recently been identified as a novel colorectal cancer antigen [117]. However, the relationship between UBXD5 and COA-1 immune response efficiency has not been studied. We believe that our analysis and review provide a new perspective for the study of UBXDF in cancer and provide new research questions for future research.

## Conclusions

We reviewed the bioinformatics and literature evidence for UBXD family in cancer. Members of the UBXD family play an important role in different types of cancer. UBXD family may affect cancer immunotherapy and drugtherapy and should be investigated in the future. Literature evidence suggests that by controlling the levels of these ubiquitin-like proteins, UBXDF may disrupt the pathways on which cancer cells rely for rapid, unchecked growth while protecting the health of normal cells. However, it remains unknown whether the remaining members of the UBXD family, such as UBXN4, UBXN11, and UBXN2B, have effects on tumour formation and progression in vitro and in vivo. More studies are needed to determine if UBXD family is a promising new target for non-genotoxic targeted therapies in treating human cancer.

**Table 3:** Summary of bioinformatics and literature evidence for UBXDF in cancers

GENE	Cancer type	Abbreviations	Clinical evidence from bioinformatics				Clinical evidence from the literature
			Expression in cancer (Additional file 1: Figure S2A)	Survival (Fig. 2A)	Stemness (Fig. 2f, g, Additional file 1: Table S2D)	Immune score (Additional file 1: Figure S3, Table S3A)	in vitro (Table 2), in vivo (Table 1), clinical, targeted medicine
FAF1	Colorectal cancer	COAD	Overexpression	NS*	Positive	Negative	Mediates apoptosis.
FAF1	Breast cancer	BRCA	Underexpression	NS	Positive	NS	Mediates migration and invasion. Mediates metastasis and invasion. Mediates migration, invasion and metastasis. Inhibits metastasis.
FAF1	Cervical cancer	CESC	–	NS	Positive	NS	Mediates tumour formation.
FAF1	Clear cell renal cell carcinoma	KIRC	Underexpression	Protective	Positive (RNAss)	Negative	Mediates growth.
FAF1	Lung cancer	LUAD and LUSC	Overexpression	NS	Positive	Negative (LUAD)	Mediates growth, migration, invasion and apoptosis.
FAF1	Gastric cancer	STAD	Overexpression	NS	Positive	Negative	Mediates cell death and apoptosis. Mediates apoptosis, proliferation and growth. Mediates apoptosis, proliferation and growth.
FAF1	Non-small cell lung carcinomas	LUAD	Overexpression	NS	Positive	Negative	Mediates proliferation, invasion, migration, cell cycle arrest and apoptosis. Reduces tumour volume and weight.
FAF1	Ovarian cancer	OV	–	NS	Positive (RNAss)	NS	Mediates cell survival.
FAF1	Prostate cancer	PRAD	NS	NS	Positive	NS	Mediates apoptosis.
FAF1	Malignant mesothelioma	MESO	–	Risk	Positive (DNAss)	NS	Inhibits Malignant mesothelioma development.
FAF1	Sarcoma	SARC	–	NS	Positive (RNAss)	Negative	A driver for leiomyosarcoma genesis.
FAF2	Lung cancers	LUAD and LUSC	Overexpression	Risk	Negative except LUAD RNAss (NS)	NS	Orlistat/Inhibits lung cancer cell proliferation and induces dramatic cell death and apoptosis.
UBXN1	BRCA1 tumour	BRCA	NS	NS	Negative (RNAss)	Positive	Regulates the enzymatic function of BRCA1.
UBXN1	Glioblastoma	LGG and GBM	NS	Protective	Positive except for LGG DNAss (negative)	Negative	Mediates growth and tumorigenesis. Inhibits tumour growth and tumorigenesis.
UBXN1	Colon adenocarcinoma	COAD	NS	NS	-	Negative	Mediates growth and tumorigenesis. Inhibits tumour growth and tumorigenesis.
UBXN1	Diffuse gliomas	LGG and GBM	NS	Protective	Positive except for LGG DNAss (negative)	Negative	Mediates proliferation and migration. Regulates protein in EGFRvIII glioma cells. Inhibits tumour progression.
UBXN1	Prostate cancer	PRAD	Overexpression	NS	NS	Positive	Mediates proliferation, migration and invasiveness
UBXN10	Colon adenocarcinoma	COAD	NS	NS	Positive (RNAss)	NS	Mediates proliferation and migration. Inhibits tumour growth.
UBXN2A	Colorectal cancer	COAD	NS	NS	Negative (DNAss)	NS	Mediates proliferation and survival. Mediates apoptosis and cell growth. Mediates cell death. Mediates apoptosis and proliferation. Inhibits tumour growth. Inhibits tumour growth. Inhibits proliferation and migration.
UBXN2A	Osteosarcoma	SARC	–	NS	NS	Negative	Mediates proliferation and migration.
UBXN7	Hepatocellular carcinoma	LIHC	Overexpression	Protective	NS	Negative	Mediates tumour formation.
UBXN7	Lung squamous cell carcinomas	LUSC	Overexpression	NS	Positive	Negative	Mediates proliferation.
UBXN8	Hepatocellular carcinoma	LIHC	Underexpression	NS	Negative	NS	Mediates cell death.

*NS not significant

### Supplementary Information

**Additional file 1: Figure S1.** Gene alteration in UBXDF in cancers.** A** UBXDF gene alterations in TCGA.** B** The OncoPrint with mutation spectrum and UBXDF gene alteration.** Figure S2. A** expression profile of cancer-noncancer tissues using TCGA individual cancer types data (noncancer tissue sample size > =5).** B** Co-expression analysis between every two genes is presented. (Blue points indicate positive correlation, while red points indicate negative correlation.)** C** K-M curve of overall survival across cancer types and UBXDF members, p <0.05.** D** Forest map shows the univariate cox regression results of UBXDF for OS.** E** UBXDF members protein expression in normal tissues.** F** UBXDF members protein expression in tumour tissues.** Figure S3.** Correlation analysis between UBXD–F expression and TME.** A**–**D** The association between UBXD–F expression and stromal score, tumour purity, ESTIMATE score and immune score in 33 TCGA cancer types. (Red points indicate positive correlation, while blue points indicate negative correlation).** Table S1.** Abbreviations and full names of nouns in the text.** Table S2. A** logFC & p value of the heatmap exhibiting the transcriptional level of the UBXDF in TCGA tumour types compared to adjacent normal tissues.** B** Co-expression network of UBXDF.** C** COX analysis between UBXDF and OS.** D**–**G** Correlation coefficient & p value of DNAss or RNAss matrix.** H** Correlation coefficient of CTRL and UBXDF.** I** Correlation coefficient of GDSC and UBXDF.** Table S3. A** Estimatescore for TCGA tumour samples.

**Additional file 2:** Confirmation of Publication and Licensing Rights.

## Data Availability

The datasets presented in this study can be found in online repositories. The names of the repository/repositories and accession number(s) can be found in the article. The datasets generated for this study are available on request to the corresponding author.

## Abbreviation

UBXDF: UBXD family
