# Supplementary material for: Exploring the role of ubiquitin regulatory X domain family proteins in cancers: bioinformatics insights, mechanisms, and implications for therapy
Source: J Transl Med. 2024 Feb 15;22:157. doi: 10.1186/s12967-024-04890-9 (PMC10870615; doi:10.1186/s12967-024-04890-9)
Supplement: Supplementary file 1 — Additional file 1: Figure S1. Gene alteration in UBXDF in cancers. A UBXDF gene alterations in TCGA. B The OncoPrint with mutation spectrum and UBXDF gene alteration. Figure S2. A expression profile of cancer-noncancer tissues using TCGA individual cancer types data (noncancer tissue sample size > =5). B Co-expression analysis between every two genes is presented. (Blue points indicate positive correlation, while red points indicate negative correlation.) C K-M curve of overall survival across cancer types and UBXDF members, p <0.05. D Forest map shows the univariate cox regression results of UBXDF for OS. E UBXDF members protein expression in normal tissues. F UBXDF members protein expression in tumour tissues. Figure S3. Correlation analysis between UBXD–F expression and TME. A–D The association between UBXD–F expression and stromal score, tumour purity, ESTIMATE score and immune score in 33 TCGA cancer types. (Red points indicate positive correlation, while blue points indicate negative correlation). Table S1. Abbreviations and full names of nouns in the text. Table S2. A logFC & p value of the heatmap exhibiting the transcriptional level of the UBXDF in TCGA tumour types compared to adjacent normal tissues. B Co-expression network of UBXDF. C COX analysis between UBXDF and OS. D–G Correlation coefficient & p value of DNAss or RNAss matrix. H Correlation coefficient of CTRL and UBXDF. I Correlation coefficient of GDSC and UBXDF. Table S3. A Estimatescore for TCGA tumour samples. [file 12967_2024_4890_MOESM1_ESM.zip › New folder/Supplementary material.docx]

Supplementary Material

# Supplementary Methods for bioinformatic analysis

## Isoform Details

The gepia2 (http://gepia2.cancer-pku.cn/#isoform) was used to explore the isoform details of UBXD family protein.

## Mutation analysis

Mutation analyses were conducted using the cBioPortal (Cerami et al., 2012). The mutation and variant data were obtained from the TCGA Pan-Cancer Atlas Studies.

## RNA-seq data analysis and plotting

The Cancer Genome Atlas (TCGA) harmonized pan-cancer dataset was obtained from the UCSC (https://xenabrowser.net/) database(Liu et al., 2018b). The full names and abbreviations of 33 types of tumours in the GDC TCGA from the UCSC Xena database are shown in **Table S1**.

For all types of TCGA tumours, ‘ggpubr’ R package (version 0.4.0) was used for differential expression analysis between tumour and adjacent tissues (Wilcox test). The difference in gene expression of the UBXD family in pan-cancer showed log2 fold change (logFC) in heatmap.

## The co-expression analysis of RNA-seq data

The ‘Corrplot’ R package (version 0.92) was used to analyze the co-expression of UBXD family. Correlation Network was performed using the OmicStudio tools (https://www.omicstudio.cn/tool) and ‘igraph’ R package (version 1.2.6).

## Cancer prognosis analysis

Using GEPIA2(http://gepia2.cancer-pku.cn), we compared the Mantel-Cox-estimated survival contribution of multiple genes across multiple cancer types(Li et al., 2021). Differences in OS outcomes between patients with high and low UBXD family expression and Kaplan-Meier curve of UBXD family genes in pan-carcinoma were analyzed. The phenotype information and survival outcome data of 33 TCGA cancer types were downloaded from the GDC TCGA dataset in the UCSC database. According to the median expression levels of UBXD family, patients were divided into a high-expression group and a low-expression group. In addition, we also performed Cox proportional hazard regression to analyze the hazard ratios of UBXD family in each TCGA tumour type.

For expression and stage analysis, GSCA(Liu et al., 2018a) used 4 types of stage (pathologic, clinical, masaoka (for THYM only), and igcccg stage (for TGCT only)) data of 9478 tumour samples from 27 cancer types (ACC, BLCA, BRCA, CESC, CHOL, COAD, DLBC, ESCA, HNSC, KICH, KIRC, KIRP, LIHC, LUAD, LUSC, MESO, OV, PAAD, READ, SKCM, STAD, TGCT, THCA, THYM, UCEC, UCS, UVM). mRNA expression and clinical stage data were merged by sample barcode. The stage subgroup must have at least 5 samples. GSCA compared the GSVA score among groups through the Wilcoxon test (number of stage groups == 2) and ANOVA test (number of stage groups > 2). Stage I includes Stage I, IA, IB and IC. Stage II includes Stage II, IIA, IIB and IIC. Stage III includes Stages III, IIIA, IIIB and IIIC. Stage IV includes Stages IV, IVA, IVB and IVC. The igcccg (The International Germ Cell Cancer Collaborative Group) stage (for TGCT only) classify samples into good (n=32), intermediate (n=9), and poor (n=2). Mann-Kendall Trend Test performed trend analysis. It is worth noting that the P value of the Mann-Kendall trend test depends on the number of research objects. Here is the number of stages (n = 4). This is too small to obtain a significant P value (< 0.05). A P value of 0.09 indicates that the gene expression continuously reduces or increases from Stage I to Stage IV. Therefore, the P value of the trend test is only for reference.

## Tumour stemness index analysis

To further analyze the association between the gene expression of the UBXDF and stemness features of pan-cancer, we used DNA methylation-based stemness index (DNAss) and mRNA expression-based stemness index (RNAss) to calculate the stemness indices of TCGA tumour samples, and according to UBXDF gene expression and stemness indices, Spearman correlation analysis was performed(Malta et al., 2018).

## The protein expression in single cell type and cancer-noncancer human tissues

The Human Protein Atlas(Sjostedt et al., 2020) (https://www.proteinatlas.org/) was used to explore the expression of UBXDF in single-cell types and normal human tissues.

## Drug-sensitive analysis

Gene expression profiles (Gene transcript level z score) for correlations analysis in NCI60 human tumour cell lines were obtained using the web-based tool provided by CellMiner(Luna et al., 2021) (version 2.2, http://discover.nci.nih.gov/cellminer/) and Pearson correlation analysis was performed based on gene expression and drug. The GSCA (Liu et al., 2018a) was used to evaluate drug-sensitive for drugs and gene expression profiles of UBXDF. Drug sensitivity and gene expression profiling data of cancer cell lines in GDSC (Yang et al., 2013) and CTRP (Rees et al., 2016) were integrated for investigation. The expression of each gene in the gene set was performed by Spearman correlation analysis with the small molecule/drug sensitivity (IC50).

## TME in pan-cancer

To study the association between the expression level of UBXD family and the proportion of immune and stromal cells in TCGA tumour types, ESTIMATE (estimation of immune scores and stromal scores)(Yoshihara et al., 2013) was utilized to reckon the proportion of these two TME components. The ESTIMATE scores were calculated according to gene expression characteristics, which reflected the purity of the tumour to a certain extent. Therefore, the Spearman correlation between the expression level of UBXD genes and stromal scores was analyzed by using the ESTIMATE package and the ‘limma’ package (version 3.50.3).

## Immune subtype analysis

In order to detect the mRNA expression levels of UBXD family in six different immune subtypes in TCGA tumour types and TCGA-SKCM type, we used Kruskal–Wallis test for differential expression analysis.

## Tumour immune infiltration analysis

The infiltrates of 24 immune cells were evaluated through ImmuCellAI(Lei et al., 2020). The GSCA (Liu et al., 2018a) was used to assess the immune infiltration of UBXDF in different cancers. The Immune infiltration & GSVA score module estimates the association between immune cells’ infiltrates and gene set expression level. The GSVA score represents the integrated level of gene set expression, which is positively correlated with the expression of the gene set. Therefore, if the GSVA score in the tumour group is higher than that in the adjacent group, it can indicate that the overall expression of the gene set in the tumour group is higher. The GSVA score was calculated through the R package ‘[GSVA](https://bmcbioinformatics.biomedcentral.com/articles/10.1186/1471-2105-14-7)’(Hanzelmann et al., 2013). The association between immune cells’ infiltrates and gene set expression level was represented by a correlation coefficient evaluated through Spearman correlation analysis. FDR adjusted the P value. The estimates of the abundance of 24 immune cell types were based on gene set signature (Document). The Immune infiltration & Gene set CNV module estimates the association between immune cells’ infiltrates and gene set CNV level. The gene set SNV represents the integrated SNV status of inputted gene set for each sample. Only when at least one gene in the input gene set is mutated in the sample is the sample classified into the mutation group. If all genes in the input gene set have no SNV in a sample, the sample is classified into the WT group. The association between immune cells’ infiltrates and gene set SNV was evaluated by comparing the mean infiltrate between gene set CNV groups through the Wilcoxon test. The P value was adjusted by FDR.

## Data Processing

All bioinformatics analyses were undertaken in R (version 4.1.2). The Wilcoxon test or Kruskal-Wallis test was employed to contrast the data from different groups. If there is no special explanation, the difference was statistically significant when the adjusted p-value was less than 0.05.

# Supplementary Figures and Tables

## Supplementary Figures


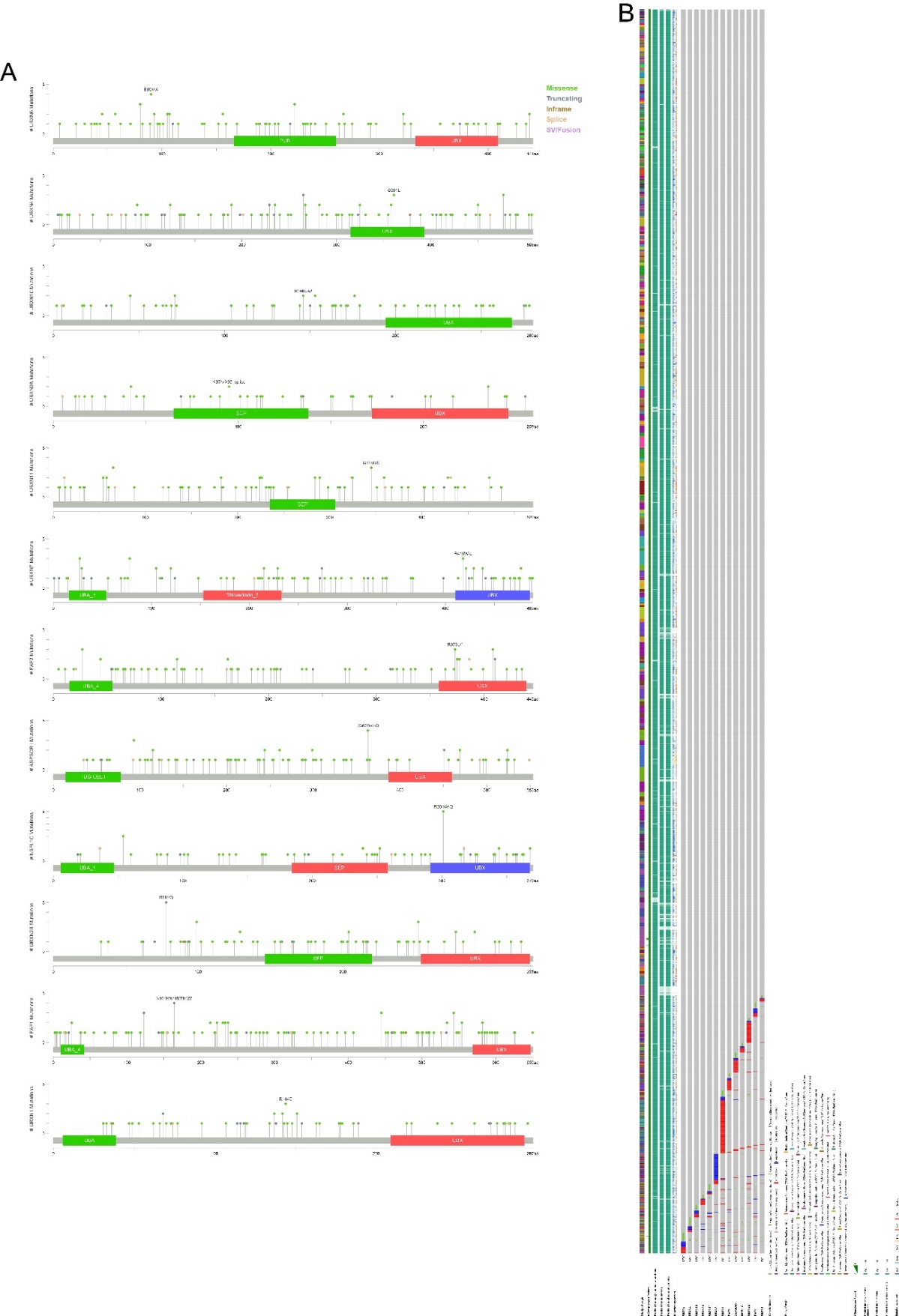


**Supplementary Figure S1.** Gene alteration in UBXDF in cancers. **(A)** UBXDF gene alterations in TCGA. **(B)** The OncoPrint with mutation spectrum and UBXDF gene alteration.


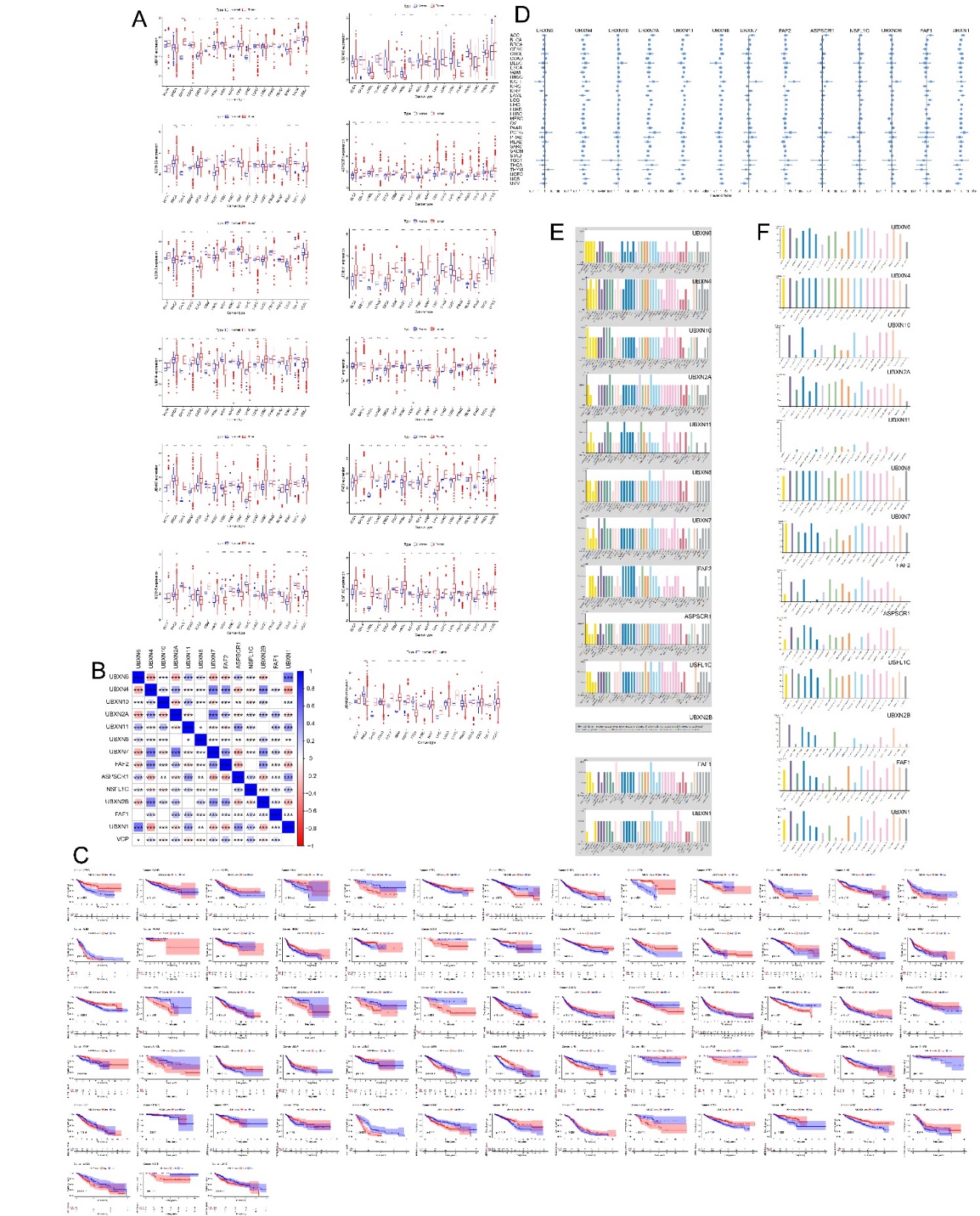


**Supplementary Figure S2** **(A)** expression profile of cancer-noncancer tissues using TCGA individual cancer types data (noncancer tissue sample size > =5). **(B)** Co-expression analysis between every two genes is presented. (Blue points indicate positive correlation, while red points indicate negative correlation.) **(C)** K-M curve of overall survival across cancer types and UBXDF members, p <0.05. **(D)** Forest map shows the univariate cox regression results of UBXDF for OS. **(E)** UBXDF members protein expression in normal tissues. **(F)** UBXDF members protein expression in tumour tissues.


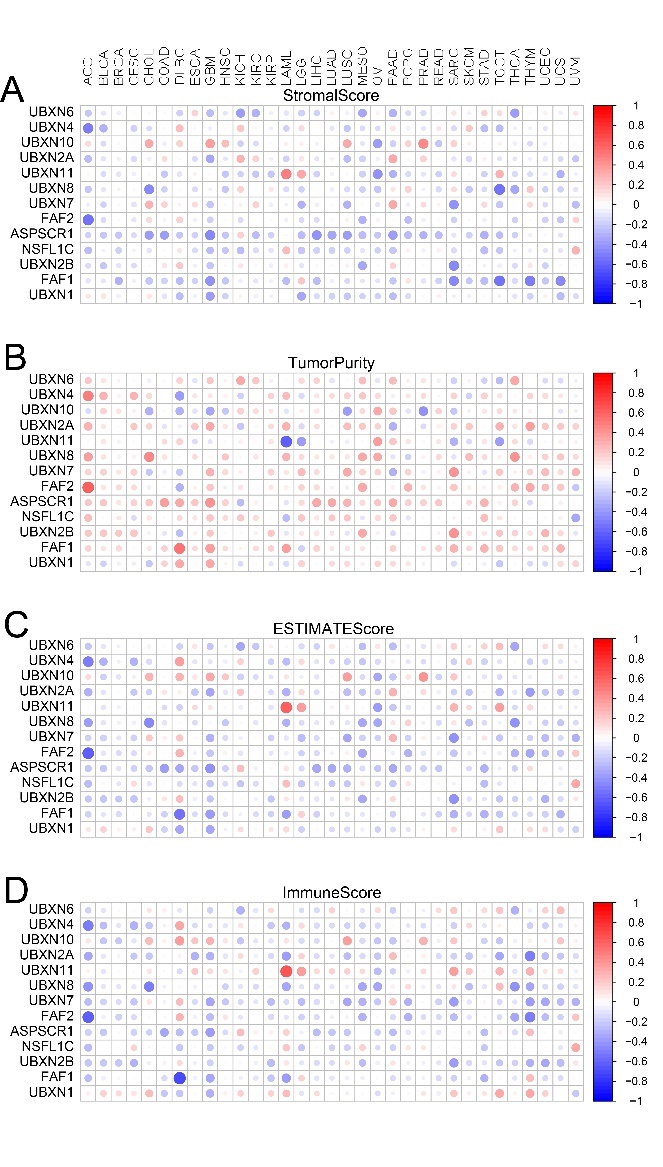


**Supplementary Figure S3** Correlation analysis between UBXD–F expression and TME. **(A-D)** The association between UBXD–F expression and stromal score, tumour purity, ESTIMATE score and immune score in 33 TCGA cancer types. (Red points indicate positive correlation, while blue points indicate negative correlation.)

## Supplementary Tables

**Supplementary Table S1** Abbreviations and full names of nouns in the text

| Shorthand | Full name |
| --- | --- |
| CNV | Copy Number Variation |
| LAML | Acute Myeloid Leukemia |
| ACC | Adrenocortical Carcinoma |
| ATLL | Adult T-cell Leukemia/Lymphoma |
| ASPSCR1 | ASPSCR1 Tether For SLC2A4, UBX Domain Containing |
| AAA | ATPases Associated with Diverse Cellular Activities |
| AAA-type ATPases | ATPases Associated with Various Cellular Activities |
| BLCA | Bladder Urothelial Carcinoma |
| LGG | Brain Lower Grade Glioma |
| BRCA | Breast Invasive Carcinoma |
| CDC48 | Cell Division Cycle 48 |
| CESC | Cervical Squamous Cell Carcinoma and Endocervical Adenocarcinoma |
| CQ | Chloroquine |
| CHOL | Cholangiocarcinoma |
| COAD | Colon Adenocarcinoma |
| COADREAD | Colon Adenocarcinoma/Rectum Adenocarcinoma Esophageal Carcinoma |
| DNAss | DNA Methylation-based Stemness Index |
| ERAD | Endoplasmic Reticulum-related Degradation |
| ESCA | Esophageal Carcinoma |
| ESCC | Esophageal Squamous Cell Carcinoma |
| ESTIMATE | Estimation of Stromal and Immune Cells in MalignantTumors Using Expression Data |
| FAF1 | Fas Associated Factor 1 |
| FAF2 | Fas Associated Factor Family Member 2 |
| GSVA | Gene Set Expression Level |
| GSCC | Gingival Squamous Cell Carcinoma |
| GBM | Glioblastoma Multiforme |
| GBMLGG | Glioma |
| HNSC | Head and Neck Squamous Cell Carcinoma |
| HTLV-1 | Human T-cell Leukemia Virus Type 1 |
| HCQ | Hydroxychloroquine |
| RIG-I | Innate Immune Receptor Retinoid-induced Gene 1 |
| KICH | Kidney Chromophobe |
| KIRC | Kidney Renal Clear Cell Carcinoma |
| KIRP | Kidney Renal Papillary Cell Carcinoma |
| LIHC | Liver Hepatocellular Carcinoma |
| LUAD | Lung Adenocarcinoma |
| LUSC | Lung Squamous Cell Carcinoma |
| DLBC | Lymphoid Neoplasm Diffuse Large B-cell Lymphoma |
| K63 | Lysine 63 |
| MESO | Mesothelioma |
| MAVS | Mitochondrial Antiviral Signaling |
| MAVs | Mitochondrial Antiviral Signaling Proteins |
| RNAss | mRNA Expression-based Stemness Index |
| NSCLC | Non-small Cell Lung Cancer |
| NS | Not Significant |
| NSFL1C | NSFL1 Cofactor |
| OV | Ovarian Serous Cystadenocarcinoma |
| OS | Overall Survival |
| PAAD | Pancreatic Adenocarcinoma |
| PDAC | Pancreatic Ductal Adenocarcinoma |
| KIPAN | Pan-kidney Cohort (KICH+KIRC+KIRP) |
| PCPG | Pheochromocytoma and Paraganglioma |
| PUB | PNGase/UBA or UBX |
| PRAD | Prostate Adenocarcinoma |
| READ | Rectum Adenocarcinoma |
| SARC | Sarcoma |
| SEP | Shp1, eyes-closed, p47 |
| SNV | Single Nucleotide Variant |
| SKCM | Skin Cutaneous Melanoma |
| STAD | Stomach Adenocarcinoma |
| STES | Stomach and Esophageal carcinoma |
| TGCT | Testicular Germ Cell Tumors |
| TβRI | TGF-β-type I Serine/Threonine Kinase Receptor |
| TβRII | TGF-β-type II Serine/Threonine Kinase Receptor |
| TCGA | The Cancer Genome Atlas |
| HPA | The Human Protein Atlas |
| THYM | Thymoma |
| THCA | Thyroid Carcinoma |
| TGF-β | Transforming Growth Factor-β |
| TME | Tumor Microenvironment |
| UBX | Ubiquitin Regulatory X |
| UBA | Ubiquitin-associated |
| UIM | Ubiquitin-interacting Motif |
| UBL | Ubiquitin-like |
| UPS | Ubiquitin-proteasome System |
| UBXD | Ubiquitin-regulated X Domain-containing Proteins |
| UBXN1 | UBX Domain Protein 1 |
| UBXN10 | UBX Domain Protein 10 |
| UBXN11 | UBX Domain Protein 11 |
| UBXN2A | UBX Domain Protein 2A |
| UBXN2B | UBX Domain Protein 2B |
| UBXN4 | UBX Domain Protein 4 |
| UBXN6 | UBX Domain Protein 6 |
| UBXN7 | UBX Domain Protein 7 |
| UBXN8 | UBX Domain Protein 8 |
| UBXDF | UBXD Family |
| UAS | Upstream Activating Sequence |
| UCS | Uterine Carcinosarcoma |
| UCEC | Uterine Corpus Endometrial Carcinoma |
| UVM | Uveal Melanoma |
| VCP/p97 | Valosin-containing Protein |
| AKT | Protein Kinase B |
| AP−26113 | Brigatinib |
| BOSC23 | Human Kidney Cell |
| CHIP | Chromatin Immunoprecipitation |
| CRL2 | Cytokine Receptor-like Factor 2 |
| CTRP | The Cancer Therapeutics Response Portal |
| CUL2 | CULLIN 2 |
| DLD-1 | Human Colorectal Adenocarcinoma Epithelial Cell |
| DU-145 | Duke University 145 |
| EGFRvIII | Epidermal growth factor receptor variant III |
| ETEA | Ubiquitin Regulatory X domain-containing Protein 8 |
| FDA | U.S.FoodandDrugAdministration |
| FDR | False discovery rate |
| FHC | Fetal Human Colon |
| GDSC | Genomics of Drug Sensitivity inCancer |
| HCC15 | Hepatocellular carcinoma 15 |
| HCC95 | Hepatocellular carcinoma 95 |
| HCT | Red Blood Cell Specific Volume |
| HCT116 | Human Colorectal Carcinoma 116 |
| HEK | Human Embryonic Kidney |
| HEK293 | Human Embryonic Kidney 293 |
| HEK293T | Human Embryonic Kidney 293T |
| Hep3B | Human Hepatocellular Carcinoma Cell |
| HepG2 | Human Hepatocellular Carcinoma Cell |
| HGC-27 | humanchorionicgonadotropin |
| HIF | Hypoxia Inducible Factor |
| HT29 | Human Colon Cancer |
| HTLV-1 | Human T-cell leukemia virus type 1 |
| HuH1 | Human Hepatocellular Carcinoma Cell |
| HuH7 | Human Hepatocellular Carcinoma Cell |
| HUVEC | Human Umbilical Vein Endothelial Cell |
| IAP | Inhibitor of Apoptosis Protein |
| IC50 | Half Maximal Inhibitory Concentration |
| IFN-γ | Interferon γ |
| IKK | Inhibitor of Kappa B Kinase |
| IL-17 | Interleukin 17 |
| LN229 | Human Brain Neuroblastoma Cell |
| LNCaP | Human Prostate Cancer Cell |
| MAIT | Mucosal-associated T cell |
| MCF7 | Michigan Cancer Foundation-7 |
| MDA-MB-231 | MD Anderson-Metastatic Breast-231 |
| U87-MG | Human Brain Astrocytoma Cell |
| MGC-803 | Human Gastric Cancer Cell |
| MM | Multiple Myeloma |
| MUL1 | Mitochondrial E3 Ubiquitin Ligase 1 |
| NEMO | NF-κB Essential Modulator |
| NF-κβ | Nuclear Factor Kappa-B |
| NK | Natural Killer Cell |
| NKT | Natural killer T cell |
| PC3 | Human Prostate Cancer Cell |
| PCR | Polymerase Chain Reaction |
| PI3K | Phosphatidylinositide 3-kinases |
| PLC | Phospholipase C |
| TβRI | Type I TGFβ Receptor |
| TβRII | Type II TGFβ Receptor |
| S181G | The Point Mutation in FAF1 |
| SLIT3 | Slit Guidance Ligand 3 |
| SMAD2/3 | SMAD family member 2/3 |
| SMAD4 | SMAD Family Member 4 |
| SMMC7721 | Human Hepatocellular Carcinoma Cell |
| SNG | Sanguinarine |
| SNU182 | Human Hepatocellular Carcinoma Cell |
| SNU387 | Human Hepatocellular Carcinoma Cell |
| SNU449 | Human Hepatocellular Carcinoma Cell |
| SPC-A1 | Human Pulmonary Adenocarcinoma Cell |
| SRD-13A | Cellosaurus cell |
| SW-480 | Human Colon Cancer Cell |
| SW48 | Human Colon Cancer Cell |
| SW620 | Human Colon Cancer Cell |
| SW900 | Human Colon Cancer Cell |
| TBK1 | TANK-binding Kinase 1 |
| TNF | Tumor Necrosis Factor |
| TP53 | Tumor Protein P53 |
| TRADD | TNF Receptor-associated Death Domain |
| TRAF2 | Tumor Necrosis Factor Receptor-associated Factor 2 |
| TRIM31 | Tripartite Motif Containing 31 |
| TRPM7 | Transient Receptor Potential Melastatin 7 |
| TUG | Glut4 Tethered Protein |
| U2OS | Human Osteosarcoma Cell |
| UPL | Domain of FAF1 |
| VHL | Von Hippel-Lindau |
| WAF1 | Wide-type53-activated Factor 1 |
| Wnt | Wingless / Integrated |
| WT | Wild Type |
| YTHDF2 | YTH Domain Family Protein 2 |

**Supplementary Table S2A** logFC&pValue of the heatmap exhibiting the transcriptional level of the UBXDF in TCGA tumour types compared to adjacent normal tissues.

**Supplementary Table S2B** Co-expression network of UBXDF.

**Supplementary Table S2C** COX analysis between UBXDF and OS.

**Supplementary Table S2D-G** Correlation coefficient & pValue of DNAss or RNAss matrix.

**Supplementary Table** **S2H** Correlation coefficient of CTRL and UBXDF.

**Supplementary Table S2I** Correlation coefficient of GDSC and UBXDF.

**Supplementary Table S3A** Estimatescore for TCGA tumour samples

# References

Cerami, E., Gao, J., Dogrusoz, U., Gross, B.E., Sumer, S.O., Aksoy, B.A., Jacobsen, A., Byrne, C.J., Heuer, M.L., Larsson, E.*, et al.* (2012). The cBio cancer genomics portal: an open platform for exploring multidimensional cancer genomics data. Cancer Discov 2, 401-404.

Hanzelmann, S., Castelo, R., and Guinney, J. (2013). GSVA: gene set variation analysis for microarray and RNA-seq data. BMC Bioinformatics 14, 7.

Lei, X., Lei, Y., Li, J.K., Du, W.X., Li, R.G., Yang, J., Li, J., Li, F., and Tan, H.B. (2020). Immune cells within the tumor microenvironment: Biological functions and roles in cancer immunotherapy. Cancer Lett 470, 126-133.

Li, C., Tang, Z., Zhang, W., Ye, Z., and Liu, F. (2021). GEPIA2021: integrating multiple deconvolution-based analysis into GEPIA. Nucleic Acids Res 49, W242-W246.

Liu, C.J., Hu, F.F., Xia, M.X., Han, L., Zhang, Q., and Guo, A.Y. (2018a). GSCALite: a web server for gene set cancer analysis. Bioinformatics 34, 3771-3772.

Liu, J., Lichtenberg, T., Hoadley, K.A., Poisson, L.M., Lazar, A.J., Cherniack, A.D., Kovatich, A.J., Benz, C.C., Levine, D.A., Lee, A.V.*, et al.* (2018b). An Integrated TCGA Pan-Cancer Clinical Data Resource to Drive High-Quality Survival Outcome Analytics. Cell 173, 400-416 e411.

Luna, A., Elloumi, F., Varma, S., Wang, Y., Rajapakse, V.N., Aladjem, M.I., Robert, J., Sander, C., Pommier, Y., and Reinhold, W.C. (2021). CellMiner Cross-Database (CellMinerCDB) version 1.2: Exploration of patient-derived cancer cell line pharmacogenomics. Nucleic Acids Res 49, D1083-D1093.

Malta, T.M., Sokolov, A., Gentles, A.J., Burzykowski, T., Poisson, L., Weinstein, J.N., Kaminska, B., Huelsken, J., Omberg, L., Gevaert, O.*, et al.* (2018). Machine Learning Identifies Stemness Features Associated with Oncogenic Dedifferentiation. Cell 173, 338-354 e315.

Rees, M.G., Seashore-Ludlow, B., Cheah, J.H., Adams, D.J., Price, E.V., Gill, S., Javaid, S., Coletti, M.E., Jones, V.L., Bodycombe, N.E.*, et al.* (2016). Correlating chemical sensitivity and basal gene expression reveals mechanism of action. Nat Chem Biol 12, 109-116.

Sjostedt, E., Zhong, W., Fagerberg, L., Karlsson, M., Mitsios, N., Adori, C., Oksvold, P., Edfors, F., Limiszewska, A., Hikmet, F.*, et al.* (2020). An atlas of the protein-coding genes in the human, pig, and mouse brain. Science 367.

Yang, W., Soares, J., Greninger, P., Edelman, E.J., Lightfoot, H., Forbes, S., Bindal, N., Beare, D., Smith, J.A., Thompson, I.R.*, et al.* (2013). Genomics of Drug Sensitivity in Cancer (GDSC): a resource for therapeutic biomarker discovery in cancer cells. Nucleic Acids Res 41, D955-961.

Yoshihara, K., Shahmoradgoli, M., Martinez, E., Vegesna, R., Kim, H., Torres-Garcia, W., Trevino, V., Shen, H., Laird, P.W., Levine, D.A.*, et al.* (2013). Inferring tumour purity and stromal and immune cell admixture from expression data. Nat Commun 4, 2612.
