# Supplementary material for: Exploring the role of ubiquitin regulatory X domain family proteins in cancers: bioinformatics insights, mechanisms, and implications for therapy
Source: J Transl Med. 2024 Feb 15;22:157. doi: 10.1186/s12967-024-04890-9 (PMC10870615; doi:10.1186/s12967-024-04890-9)
Supplement: Supplementary file 1 — Additional file 1: Figure S1. Gene alteration in UBXDF in cancers. A UBXDF gene alterations in TCGA. B The OncoPrint with mutation spectrum and UBXDF gene alteration. Figure S2. A expression profile of cancer-noncancer tissues using TCGA individual cancer types data (noncancer tissue sample size > =5). B Co-expression analysis between every two genes is presented. (Blue points indicate positive correlation, while red points indicate negative correlation.) C K-M curve of overall survival across cancer types and UBXDF members, p <0.05. D Forest map shows the univariate cox regression results of UBXDF for OS. E UBXDF members protein expression in normal tissues. F UBXDF members protein expression in tumour tissues. Figure S3. Correlation analysis between UBXD–F expression and TME. A–D The association between UBXD–F expression and stromal score, tumour purity, ESTIMATE score and immune score in 33 TCGA cancer types. (Red points indicate positive correlation, while blue points indicate negative correlation). Table S1. Abbreviations and full names of nouns in the text. Table S2. A logFC & p value of the heatmap exhibiting the transcriptional level of the UBXDF in TCGA tumour types compared to adjacent normal tissues. B Co-expression network of UBXDF. C COX analysis between UBXDF and OS. D–G Correlation coefficient & p value of DNAss or RNAss matrix. H Correlation coefficient of CTRL and UBXDF. I Correlation coefficient of GDSC and UBXDF. Table S3. A Estimatescore for TCGA tumour samples. [file 12967_2024_4890_MOESM1_ESM.zip › New folder/Table S1.docx]

**Supplementary Table S1. Abbreviations and full names of nouns in the text**

| Shorthand | Full name |
| --- | --- |
| CNV | Copy Number Variation |
| LAML | Acute Myeloid Leukemia |
| ACC | Adrenocortical Carcinoma |
| ATLL | Adult T-cell Leukemia/Lymphoma |
| ASPSCR1 | ASPSCR1 Tether For SLC2A4, UBX Domain Containing |
| AAA | ATPases Associated with Diverse Cellular Activities |
| AAA-type ATPases | ATPases Associated with Various Cellular Activities |
| BLCA | Bladder Urothelial Carcinoma |
| LGG | Brain Lower Grade Glioma |
| BRCA | Breast Invasive Carcinoma |
| CDC48 | Cell Division Cycle 48 |
| CESC | Cervical Squamous Cell Carcinoma and Endocervical Adenocarcinoma |
| CQ | Chloroquine |
| CHOL | Cholangiocarcinoma |
| COAD | Colon Adenocarcinoma |
| COADREAD | Colon Adenocarcinoma/Rectum Adenocarcinoma Esophageal Carcinoma |
| DNAss | DNA Methylation-based Stemness Index |
| ERAD | Endoplasmic Reticulum-related Degradation |
| ESCA | Esophageal Carcinoma |
| ESCC | Esophageal Squamous Cell Carcinoma |
| ESTIMATE | Estimation of Stromal and Immune Cells in MalignantTumors Using Expression Data |
| FAF1 | Fas Associated Factor 1 |
| FAF2 | Fas Associated Factor Family Member 2 |
| GSVA | Gene Set Expression Level |
| GSCC | Gingival Squamous Cell Carcinoma |
| GBM | Glioblastoma Multiforme |
| GBMLGG | Glioma |
| HNSC | Head and Neck Squamous Cell Carcinoma |
| HTLV-1 | Human T-cell Leukemia Virus Type 1 |
| HCQ | Hydroxychloroquine |
| RIG-I | Innate Immune Receptor Retinoid-induced Gene 1 |
| KICH | Kidney Chromophobe |
| KIRC | Kidney Renal Clear Cell Carcinoma |
| KIRP | Kidney Renal Papillary Cell Carcinoma |
| LIHC | Liver Hepatocellular Carcinoma |
| LUAD | Lung Adenocarcinoma |
| LUSC | Lung Squamous Cell Carcinoma |
| DLBC | Lymphoid Neoplasm Diffuse Large B-cell Lymphoma |
| K63 | Lysine 63 |
| MESO | Mesothelioma |
| MAVS | Mitochondrial Antiviral Signaling |
| MAVs | Mitochondrial Antiviral Signaling Proteins |
| RNAss | mRNA Expression-based Stemness Index |
| NSCLC | Non-small Cell Lung Cancer |
| NS | Not Significant |
| NSFL1C | NSFL1 Cofactor |
| OV | Ovarian Serous Cystadenocarcinoma |
| OS | Overall Survival |
| PAAD | Pancreatic Adenocarcinoma |
| PDAC | Pancreatic Ductal Adenocarcinoma |
| KIPAN | Pan-kidney Cohort (KICH+KIRC+KIRP) |
| PCPG | Pheochromocytoma and Paraganglioma |
| PUB | PNGase/UBA or UBX |
| PRAD | Prostate Adenocarcinoma |
| READ | Rectum Adenocarcinoma |
| SARC | Sarcoma |
| SEP | Shp1, eyes-closed, p47 |
| SNV | Single Nucleotide Variant |
| SKCM | Skin Cutaneous Melanoma |
| STAD | Stomach Adenocarcinoma |
| STES | Stomach and Esophageal carcinoma |
| TGCT | Testicular Germ Cell Tumors |
| TβRI | TGF-β-type I Serine/Threonine Kinase Receptor |
| TβRII | TGF-β-type II Serine/Threonine Kinase Receptor |
| TCGA | The Cancer Genome Atlas |
| HPA | The Human Protein Atlas |
| THYM | Thymoma |
| THCA | Thyroid Carcinoma |
| TGF-β | Transforming Growth Factor-β |
| TME | Tumor Microenvironment |
| UBX | Ubiquitin Regulatory X |
| UBA | Ubiquitin-associated |
| UIM | Ubiquitin-interacting Motif |
| UBL | Ubiquitin-like |
| UPS | Ubiquitin-proteasome System |
| UBXD | Ubiquitin-regulated X Domain-containing Proteins |
| UBXN1 | UBX Domain Protein 1 |
| UBXN10 | UBX Domain Protein 10 |
| UBXN11 | UBX Domain Protein 11 |
| UBXN2A | UBX Domain Protein 2A |
| UBXN2B | UBX Domain Protein 2B |
| UBXN4 | UBX Domain Protein 4 |
| UBXN6 | UBX Domain Protein 6 |
| UBXN7 | UBX Domain Protein 7 |
| UBXN8 | UBX Domain Protein 8 |
| UBXDF | UBXD Family |
| UAS | Upstream Activating Sequence |
| UCS | Uterine Carcinosarcoma |
| UCEC | Uterine Corpus Endometrial Carcinoma |
| UVM | Uveal Melanoma |
| VCP/p97 | Valosin-containing Protein |
| AKT | Protein Kinase B |
| AP−26113 | Brigatinib |
| BOSC23 | Human Kidney Cell |
| CHIP | Chromatin Immunoprecipitation |
| CRL2 | Cytokine Receptor-like Factor 2 |
| CTRP | The Cancer Therapeutics Response Portal |
| CUL2 | CULLIN 2 |
| DLD-1 | Human Colorectal Adenocarcinoma Epithelial Cell |
| DU-145 | Duke University 145 |
| EGFRvIII | Epidermal growth factor receptor variant III |
| ETEA | Ubiquitin Regulatory X domain-containing Protein 8 |
| FDA | U.S.FoodandDrugAdministration |
| FDR | False discovery rate |
| FHC | Fetal Human Colon |
| GDSC | Genomics of Drug Sensitivity inCancer |
| HCC15 | Hepatocellular carcinoma 15 |
| HCC95 | Hepatocellular carcinoma 95 |
| HCT | Red Blood Cell Specific Volume |
| HCT116 | Human Colorectal Carcinoma 116 |
| HEK | Human Embryonic Kidney |
| HEK293 | Human Embryonic Kidney 293 |
| HEK293T | Human Embryonic Kidney 293T |
| Hep3B | Human Hepatocellular Carcinoma Cell |
| HepG2 | Human Hepatocellular Carcinoma Cell |
| HGC-27 | humanchorionicgonadotropin |
| HIF | Hypoxia Inducible Factor |
| HT29 | Human Colon Cancer |
| HTLV-1 | Human T-cell leukemia virus type 1 |
| HuH1 | Human Hepatocellular Carcinoma Cell |
| HuH7 | Human Hepatocellular Carcinoma Cell |
| HUVEC | Human Umbilical Vein Endothelial Cell |
| IAP | Inhibitor of Apoptosis Protein |
| IC50 | Half Maximal Inhibitory Concentration |
| IFN-γ | Interferon γ |
| IKK | Inhibitor of Kappa B Kinase |
| IL-17 | Interleukin 17 |
| LN229 | Human Brain Neuroblastoma Cell |
| LNCaP | Human Prostate Cancer Cell |
| MAIT | Mucosal-associated T cell |
| MCF7 | Michigan Cancer Foundation-7 |
| MDA-MB-231 | MD Anderson-Metastatic Breast-231 |
| U87-MG | Human Brain Astrocytoma Cell |
| MGC-803 | Human Gastric Cancer Cell |
| MM | Multiple Myeloma |
| MUL1 | Mitochondrial E3 Ubiquitin Ligase 1 |
| NEMO | NF-κB Essential Modulator |
| NF-κβ | Nuclear Factor Kappa-B |
| NK | Natural Killer Cell |
| NKT | Natural killer T cell |
| PC3 | Human Prostate Cancer Cell |
| PCR | Polymerase Chain Reaction |
| PI3K | Phosphatidylinositide 3-kinases |
| PLC | Phospholipase C |
| TβRI | Type I TGFβ Receptor |
| TβRII | Type II TGFβ Receptor |
| S181G | The Point Mutation in FAF1 |
| SLIT3 | Slit Guidance Ligand 3 |
| SMAD2/3 | SMAD family member 2/3 |
| SMAD4 | SMAD Family Member 4 |
| SMMC7721 | Human Hepatocellular Carcinoma Cell |
| SNG | Sanguinarine |
| SNU182 | Human Hepatocellular Carcinoma Cell |
| SNU387 | Human Hepatocellular Carcinoma Cell |
| SNU449 | Human Hepatocellular Carcinoma Cell |
| SPC-A1 | Human Pulmonary Adenocarcinoma Cell |
| SRD-13A | Cellosaurus cell |
| SW-480 | Human Colon Cancer Cell |
| SW48 | Human Colon Cancer Cell |
| SW620 | Human Colon Cancer Cell |
| SW900 | Human Colon Cancer Cell |
| TBK1 | TANK-binding Kinase 1 |
| TNF | Tumor Necrosis Factor |
| TP53 | Tumor Protein P53 |
| TRADD | TNF Receptor-associated Death Domain |
| TRAF2 | Tumor Necrosis Factor Receptor-associated Factor 2 |
| TRIM31 | Tripartite Motif Containing 31 |
| TRPM7 | Transient Receptor Potential Melastatin 7 |
| TUG | Glut4 Tethered Protein |
| U2OS | Human Osteosarcoma Cell |
| UPL | Domain of FAF1 |
| VHL | Von Hippel-Lindau |
| WAF1 | Wide-type53-activated Factor 1 |
| Wnt | Wingless / Integrated |
| WT | Wild Type |
| YTHDF2 | YTH Domain Family Protein 2 |
